# Supplementary material for: Association of coincident self-reported mental health problems and alcohol intake with all-cause and cardiovascular disease mortality: A Norwegian pooled population analysis
Source: PLoS Med. 2020 Feb 3;17(2):e1003030. doi: 10.1371/journal.pmed.1003030 (PMC6996806; doi:10.1371/journal.pmed.1003030)
Supplement: S1 Checklist — (DOCX) [file pmed.1003030.s001.docx]

**The RECORD statement – checklist of items, extended from the STROBE statement, that should be reported in observational studies using routinely collected health data.**

|  | **Item No.** | **STROBE items** | **Location in manuscript where items are reported** | **RECORD items** | **Location in manuscript where items are reported** |
| --- | --- | --- | --- | --- | --- |
| **Title and abstract** | | | | | |
|  | 1 | (a) Indicate the study’s design with a commonly used term in the title or the abstract (b) Provide in the abstract an informative and balanced summary of what was done and what was found | a) Pooled analysis of Norwegian surveys  b) Yes. | RECORD 1.1: The type of data used should be specified in the title or abstract. When possible, the name of the databases used should be included.  RECORD 1.2: If applicable, the geographic region and timeframe within which the study took place should be reported in the title or abstract.  RECORD 1.3: If linkage between databases was conducted for the study, this should be clearly stated in the title or abstract. | - 1. Pooled analysis of Norwegian surveys.   2. Norway is mentioned as well as the time the surveys were performed and the time period they were followed prospectively for all-cause and CVD events.   3. We state in the abstract that the participants were followed prospectively via data linkage to national registries. |
| **Introduction** | | | | | |
| Background rationale | 2 | Explain the scientific background and rationale for the investigation being reported | First, second, and third paragraph |  |  |
| Objectives | 3 | State specific objectives, including any prespecified hypotheses | Third paragraph |  |  |
| **Methods** | | | | | |
| Study Design | 4 | Present key elements of study design early in the paper | Given in paragraph “Study population, data linkage, ethical approval, and selection”. |  |  |
| Setting | 5 | Describe the setting, locations, and relevant dates, including periods of recruitment, exposure, follow-up, and data collection | Given in paragraph “Study population, data linkage, ethical approval, and selection”.  Table 1 provides an overview of the surveys constituting the source population.  Details on exposure, covariates, and outcomes are presented in separate paragraphs in the method section. |  |  |
| Participants | 6 | *(a) Cohort study* - Give the eligibility criteria, and the sources and methods of selection of participants. Describe methods of follow-up  *Case-control study* - Give the eligibility criteria, and the sources and methods of case ascertainment and control selection. Give the rationale for the choice of cases and controls  *Cross-sectional study* - Give the eligibility criteria, and the sources and methods of selection of participants  *(b) Cohort study* - For matched studies, give matching criteria and number of exposed and unexposed  *Case-control study* - For matched studies, give matching criteria and the number of controls per case | a) Given in paragraph “Study population, data linkage, ethical approval, and selection”.  Also given in the paragraph “Outcome”. | RECORD 6.1: The methods of study population selection (such as codes or algorithms used to identify subjects) should be listed in detail. If this is not possible, an explanation should be provided.  RECORD 6.2: Any validation studies of the codes or algorithms used to select the population should be referenced. If validation was conducted for this study and not published elsewhere, detailed methods and results should be provided.  RECORD 6.3: If the study involved linkage of databases, consider use of a flow diagram or other graphical display to demonstrate the data linkage process, including the number of individuals with linked data at each stage. | 6.1. We provide an explanation on how we selected the study population from the source population in the paragraph “Study population, data linkage, ethical approval, and selection”. Programming code is provided in “S2 Text”  6.2 Not applicable.  6.3. The personal identification number in Norway provides almost complete data linkage to national registries, so the need to highlight the number of individuals linked is not necessary. |
| Variables | 7 | Clearly define all outcomes, exposures, predictors, potential confounders, and effect modifiers. Give diagnostic criteria, if applicable. | Defined in paragraphs: “Alcohol consumption”, “Mental health index”, “Covariates”, “Outcome”, and in “Statistical analyses” | RECORD 7.1: A complete list of codes and algorithms used to classify exposures, outcomes, confounders, and effect modifiers should be provided. If these cannot be reported, an explanation should be provided. | Programming code is provided in “S2 Text” |
| Data sources/ measurement | 8 | For each variable of interest, give sources of data and details of methods of assessment (measurement).  Describe comparability of assessment methods if there is more than one group | Defined in paragraphs: “Alcohol consumption”, “Mental health index”, “Covariates”, “Outcome”, and in “Statistical analyses” |  |  |
| Bias | 9 | Describe any efforts to address potential sources of bias | Described in discussion in paragraph “Methodological considerations” |  |  |
| Study size | 10 | Explain how the study size was arrived at | Given in paragraph “Study population, data linkage, ethical approval, and selection” and in table S1, and in Fig-1 Flow chart, and in paragraph “Study population”. |  |  |
| Quantitative variables | 11 | Explain how quantitative variables were handled in the analyses. If applicable, describe which groupings were chosen, and why | Defined in paragraphs: “Alcohol consumption”, “Mental health index”, “Covariates”, “Outcome”, and in “Statistical analyses” |  |  |
| Statistical methods | 12 | (a) Describe all statistical methods, including those used to control for confounding  (b) Describe any methods used to examine subgroups and interactions  (c) Explain how missing data were addressed  (d) *Cohort study* - If applicable, explain how loss to follow-up was addressed  *Case-control study* - If applicable, explain how matching of cases and controls was addressed  *Cross-sectional study* - If applicable, describe analytical methods taking account of sampling strategy  (e) Describe any sensitivity analyses | Described in paragraph: “Study population, data linkage, ethical approval, and selection” and in “Statistical analysis” |  |  |
| Data access and cleaning methods |  | .. |  | RECORD 12.1: Authors should describe the extent to which the investigators had access to the database population used to create the study population.  RECORD 12.2: Authors should provide information on the data cleaning methods used in the study. | 12.1. Described in paragraph: “Study population, data linkage, ethical approval, and selection”. It emerges that the authors created the study population based on de-identified data from the different health surveys.  12.2. We have explained in the manuscript text in the paragraph “Alcohol consumption” as well as in “Table 1” how we harmonised data on alcohol intake. Additional information for other variables can be derived from “S2 Text”. |
| Linkage |  | .. |  | RECORD 12.3: State whether the study included person-level, institutional-level, or other data linkage across two or more databases. The methods of linkage and methods of linkage quality evaluation should be provided. | Described in paragraph: “Study population, data linkage, ethical approval, and selection”. It emerges that we are using person-level data from health surveys. The data linkage method is explained and we state that this gives nearly complete linkage. |
| **Results** | | | | | |
| Participants | 13 | (a) Report the numbers of individuals at each stage of the study (*e.g.*, numbers potentially eligible, examined for eligibility, confirmed eligible, included in the study, completing follow-up, and analysed)  (b) Give reasons for non-participation at each stage.  (c) Consider use of a flow diagram | Described in paragraph: “Study population”. A flow chart is provided as a supplemental figure (S1). List of surveys in table S1. | RECORD 13.1: Describe in detail the selection of the persons included in the study (*i.e.,* study population selection) including filtering based on data quality, data availability and linkage. The selection of included persons can be described in the text and/or by means of the study flow diagram. | 13.1. Described in paragraph: “Study population”. A flow chart is provided as a supplemental figure (S1). List of surveys in table S1. List of codes in “S2 Text”. |
| Descriptive data | 14 | (a) Give characteristics of study participants (*e.g.*, demographic, clinical, social) and information on exposures and potential confounders  (b) Indicate the number of participants with missing data for each variable of interest  (c) *Cohort study* - summarise follow-up time (*e.g.*, average and total amount) | Described in results in paragraph: “Descriptive statistics” and “Outcome statistics and crude survival”, including tables TABLE S2 and S3, and also a list of surveys in TABLE S1. |  |  |
| Outcome data | 15 | *Cohort study* - Report numbers of outcome events or summary measures over time  *Case-control study* - Report numbers in each exposure category, or summary measures of exposure  *Cross-sectional study* - Report numbers of outcome events or summary measures | Described in paragraph: “Outcome statistics and crude survival” and given for each group of average alcohol intake and mental health index in TABLE 1 and TABLE 2. |  |  |
| Main results | 16 | (a) Give unadjusted estimates and, if applicable, confounder-adjusted estimates and their precision (e.g., 95% confidence interval). Make clear which confounders were adjusted for and why they were included  (b) Report category boundaries when continuous variables were categorized  (c) If relevant, consider translating estimates of relative risk into absolute risk for a meaningful time period | Presented in results in paragraph: “Mental health problems” and in paragraph “Alcohol intake” and in paragraph “Combined assessment of mental health problems and alcohol intake”. Also given in TABLE 1-2 and TABLE S4 and S5.  Because of the uneven distribution of age and sex according to the exposure, we consider completely unadjusted estimates to be less informative, and consider age and sex adjusted estimates to be the crude estimate. |  |  |
| Other analyses | 17 | Report other analyses done—e.g., analyses of subgroups and interactions, and sensitivity analyses | Subgroups and interactions is part of the main analyses. Reported in “Statistical analyses”. |  |  |
| **Discussion** | | | | | |
| Key results | 18 | Summarise key results with reference to study objectives | Discussed sequentially in the beginning of three paragraphs in the discussion: “Mental health and mortality”m and “Alcohol intake and mortality” and then in “Co-occurrence of mental health problems and alcohol intake and mortality” |  |  |
| Limitations | 19 | Discuss limitations of the study, taking into account sources of potential bias or imprecision. Discuss both direction and magnitude of any potential bias | Discussed in paragraph “Methodological considerations”, but also in the other paragraphs in the discussion | RECORD 19.1: Discuss the implications of using data that were not created or collected to answer the specific research question(s). Include discussion of misclassification bias, unmeasured confounding, missing data, and changing eligibility over time, as they pertain to the study being reported. | 19.1. This is data was collected to answer research questions as well as to monitor the cardiovascular health of Norwegians. Nevertheless, we discuss different sources of bias in the paragraph “Methodological considerations” in the discussion. Missing data is also presented in the result section in the paragraph “Study population”. |
| Interpretation | 20 | Give a cautious overall interpretation of results considering objectives, limitations, multiplicity of analyses, results from similar studies, and other relevant evidence | Given in each paragraph in the discussion, following the key results. |  |  |
| Generalisability | 21 | Discuss the generalisability (external validity) of the study results | Discussed in the paragraph “Methodological considerations”, especially in the last part. |  |  |
| **Other Information** | | | | | |
| Funding | 22 | Give the source of funding and the role of the funders for the present study and, if applicable, for the original study on which the present article is based | Provided as a separate statement and not in the manuscript text. |  |  |
| Accessibility of protocol, raw data, and programming code |  | .. | Protocol included as supplemental data “S1 Protocol”. Data access statement provides information regarding access to the raw data. Programming code is provided in “S2 Text” | RECORD 22.1: Authors should provide information on how to access any supplemental information such as the study protocol, raw data, or programming code. | 22.1. Protocol included as supplemental data “S1 Protocol”. Data access statement provides information regarding access to the raw data.  Programming code is provided in “S2 Text” |

*Reference: Benchimol EI, Smeeth L, Guttmann A, Harron K, Moher D, Petersen I, Sørensen HT, von Elm E, Langan SM, the RECORD Working Committee. The REporting of studies Conducted using Observational Routinely-collected health Data (RECORD) Statement. *PLoS Medicine* 2015; in press.

*Checklist is protected under Creative Commons Attribution ([CC BY](http://creativecommons.org/licenses/by/4.0/)) license.
