# Supplementary figures and images for: Association of coincident self-reported mental health problems and alcohol intake with all-cause and cardiovascular disease mortality: A Norwegian pooled population analysis
Source: PLoS Med. 2020 Feb 3;17(2):e1003030. doi: 10.1371/journal.pmed.1003030 (PMC6996806; doi:10.1371/journal.pmed.1003030)

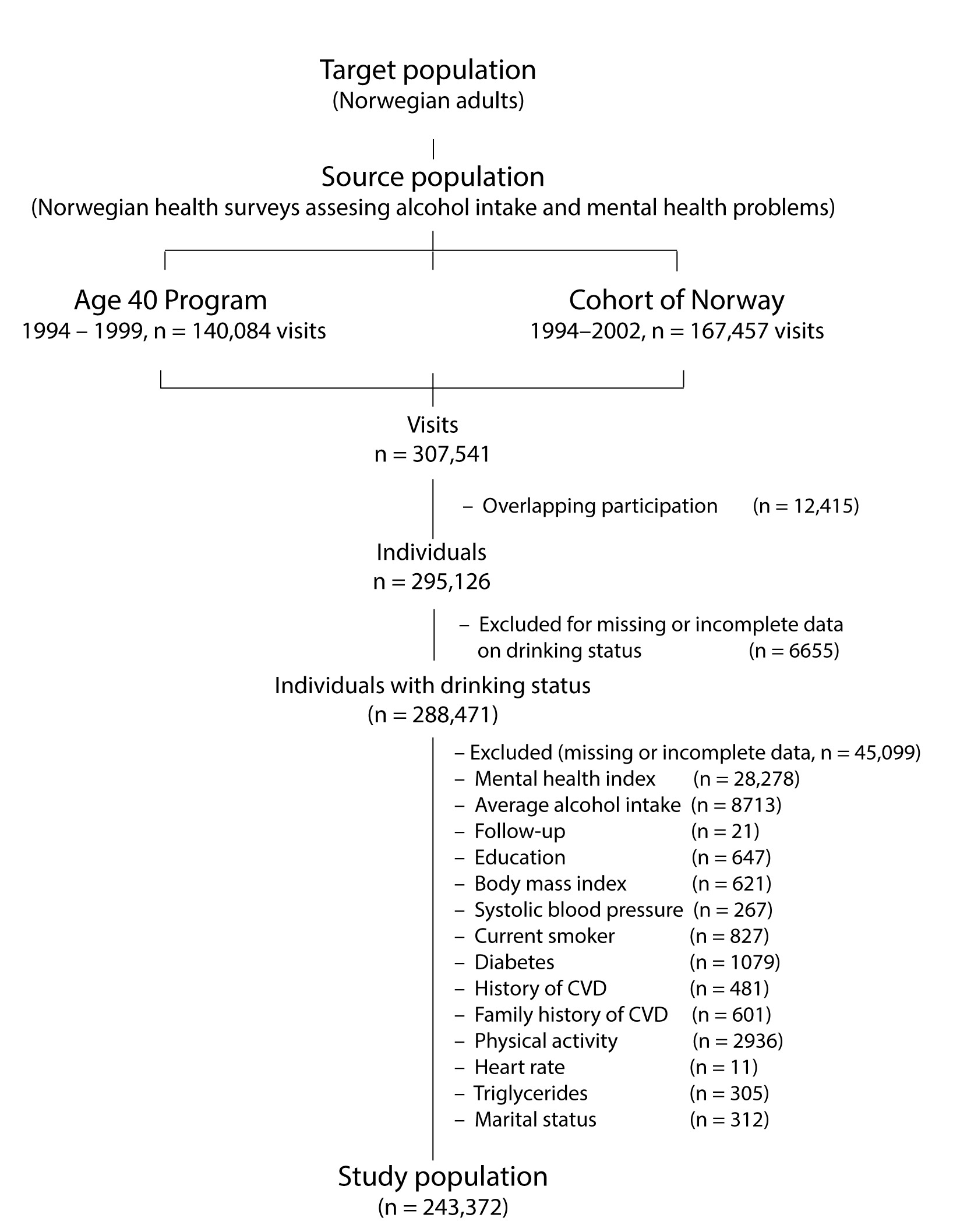

Supplement: S1 Fig — (TIF) [file pmed.1003030.s002.tif]

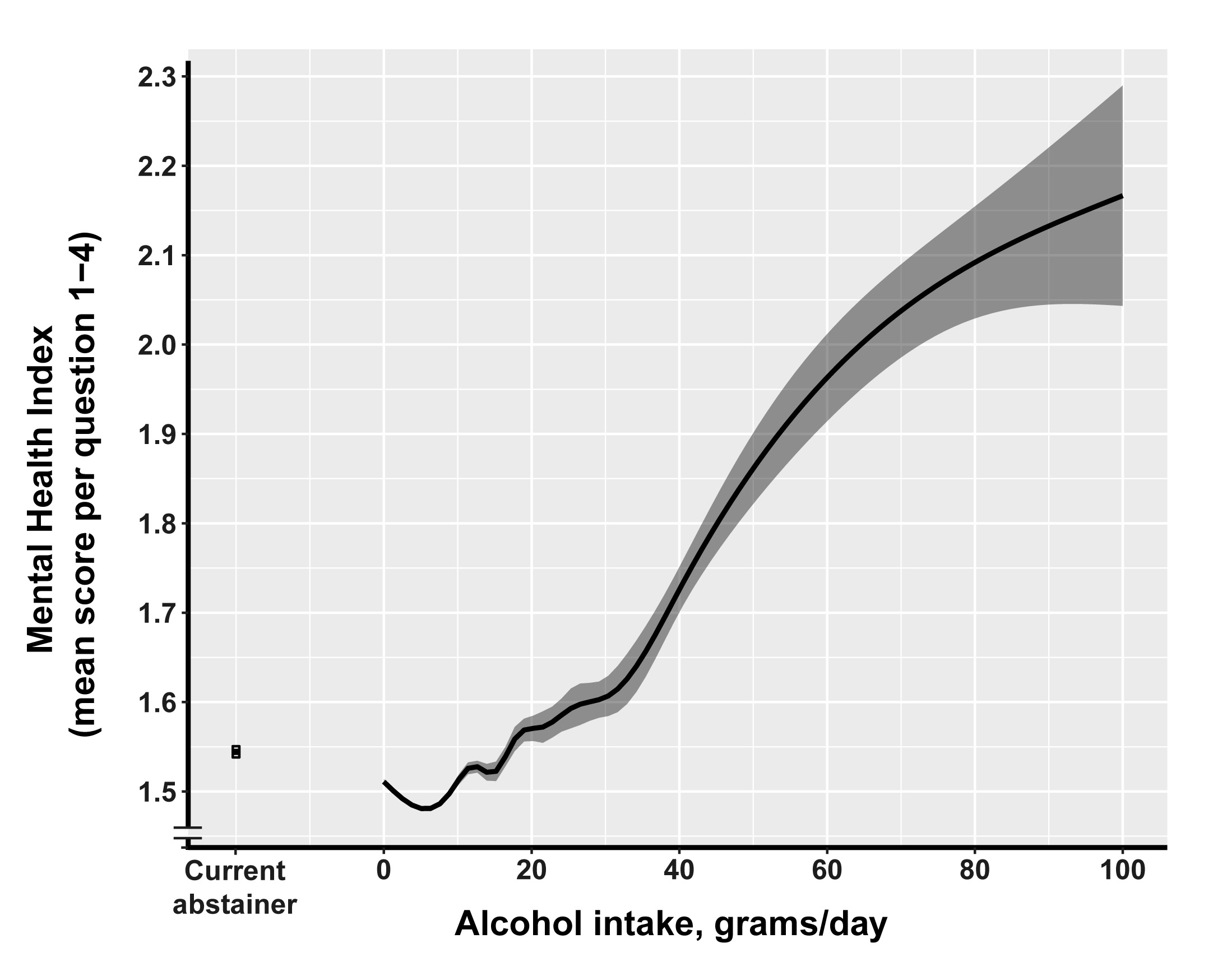

Supplement: S2 Fig — The boxplot depicts the mean value of the mental health index with 95% confidence intervals among current abstainers. The curve depicts the smoothed mean of the mental health index according to the average alcohol intake (grams/day) among current drinkers. The grey shading depicts the 95% confidence intervals. The vertical axis has been truncated. (TIF) [file pmed.1003030.s003.tif]
