# Supplementary material for: Association of coincident self-reported mental health problems and alcohol intake with all-cause and cardiovascular disease mortality: A Norwegian pooled population analysis
Source: PLoS Med. 2020 Feb 3;17(2):e1003030. doi: 10.1371/journal.pmed.1003030 (PMC6996806; doi:10.1371/journal.pmed.1003030)
Supplement: S1 Protocol — (PDF) [file pmed.1003030.s004.pdf]

Risk and benefit of combined moderate and binge drinking on cardiovascular diseases (CVD)

Objectives

An overall aim of the project is to assess the possible protective effect of moderate alcohol consumption against CVD and which factors moderates such an effect. The study will benefit from a large sample size which will enable us to investigate subgroups of alcohol consumption pattern in more detail than previously done in research.

Research questions (RQ)

(1) To investigate the risk and protective effects on CVD morbidity and mortality associated with various combinations of moderate alcohol consumption and binge drinking. We will here also look at risk in the sub-group of patients with pre-existing CVD.

(2) To investigate if the effects of moderate alcohol consumption and binge drinking on CVD can be explained by or interact with mental distress (depression and/or anxiety).

(3) To investigate if the effects of moderate alcohol consumption and binge drinking on CVD can be explained by or interact with cognitive ability (IQ), educational level, or other socioeconomic factors.

(4) First, to investigate how alcohol consumption in family members are related to CVD risk among index individuals. Secondly, to use alcohol consumption among siblings and offspring as instrumental variable to assess the causal effects of alcohol consumption pattern on CVD.

(5) Using pairs of siblings, monozygotic and dizygotic twins discordant for CVD to estimating the association between moderate alcohol and binge drinking and CVD *within families*. These results will adjust for early life and family factors.

1. Relevance to the call for proposals:

The health effect of alcohol consumption pattern (moderate and binge drinking) is of significant public health relevance because alcohol consumption in Norway and other countries in Northern Europe have been increasing since 2000.[1] Furthermore, the consumption pattern is changing from binge drinking towards moderate regular intake.[17] Norway is in a fortunate position to address this issue because large scale health surveys with data on both moderate and binge drinking have been linked to a multigenerational database, hospital discharge on CVD morbidity and mortality and other registries.

The national policy building upon a premise that total consumption affects number of problem drinkers might be under pressure. An implication of this principle of solidarity is that anyone can potentially experience health problems due to alcohol consumption. However, the scientific evidence base for population wide approaches is challenged for several reasons: First, the effect of alcohol consumption on CVD appears to be divergent (RQ 1). But also, alcohol consumption might be influenced by underlying factors, which calls for a reconsidering on the issue of human agency in policy formulation. Individual and personal

characteristics, such as psychosocial and cognitive, might explain a large and increasing part of the variation in health behavior (RQ 2&3). It has also been suggested that personal characteristics to an increasing extent explain socioeconomic inequalities in health. [2] A better understanding of vulnerable subgroups in modern society where according to social theorists, the individual is put at an increased responsibility for managing their own health risk,[3] is imperative for developing future health and alcohol policy. And finally, both alcohol consumption and CVD risk tend to be clustered within families (RQ 4&5). This could indicate the scope for early prevention of both harmful alcohol use and CVD.

2. Aspects relating to the research project

Background and status of knowledge

Alcohol has a negative effect on many health-related outcomes but for heart diseases the effects seem to be mixed.[1] Alcohol use is negatively related to hypertensive disease,[4] hemorrhagic stroke[5] and atrial fibrillation.[6] For ischemic heart disease (IHD) and ischemic stroke (cardiovascular diseases, CVD), the relationship is complex. Chronic heavy alcohol use has been associated with adverse cardiovascular outcomes.[7] But light to moderate drinking seems to be related to reduced risk of ischemic diseases.[8]

It is not well understood to what extent frequent episodes of heavy alcohol consumption may counteract the beneficial effect on heart disease and by which mechanisms episodes of heavy alcohol have a negative effect on heart disease. Even rare and occasional episodes of binge drinking seem to increase the risk in a recent study from the northernmost part of Norway.[21] One would expect the protective effect to be more pronounced in individuals who do not binge drink, and not present in individuals who report substantial binge drinking. A recent meta-analysis on binge drinking including only 4718 IHD events gave some support for this notion. [9] Our study will add to this by 6500 CVD cases.

Norwegian studies on adolescent and adult abstainers have shown higher risk of psychosocial factors and might suggest a J-shaped pattern.[17,11] A general finding is that abstainers tend to have worse health and are poorer.[10] One interpretation of this and similar findings is that since alcohol is such an important token in social interaction, abstainers are likely to differ as a social group and have a wide range of individual characteristics that could put them at increased risk of several health problems.[11] Those who have moderate intake have in studies been shown to have higher cognitive ability compared to abstainers and that heavy drinkers have lower cognitive ability. [12;13]

But there is surprisingly little evidence on how the CVD risk associated with alcohol interact with psychosocial factors, such as cognitive ability and mental distress.[14] An editorial stated that: "….the area of research is considered fragmented to the extreme…conducted in a wide range of disciplines (e.g. anthropology, biology, chemistry, economics, history, law, medicine, neurology, psychology, sociology)".[15] Thus, plausible explanations on effects are theoretically grounded in several fields of knowledge. Our project and team aims explicitly to exploit this interdisciplinary challenge.

We recently showed that a substantial part of the educational gradient in both CVD and alcohol related mortality could be explained by family factors early in life shared by siblings.[16] This could indicate that risk factors for CVD and alcohol use share common origin from childhood onwards, and that some of the estimated effect size both for moderate and

binge drinking may be explained by early life confounding or family factors.[18] Few family and life course studies have investigated alcohol and CVD jointly.

Approaches, hypothesis and choice of method

The study has some important strengths: the sample size and comprehensive data linkage with family data, the availability of information on both moderate and binge drinking, reasonably long follow-up and linkage to information on both morbidity and mortality. Using morbidity data on hospital discharge on incidences of CVD increases the number of cases by a factor of approximately three to four compared to mortality data, and further increases statistical power further (RQ 1). This enables us to investigate combinations of moderate alcohol consumption and binge drinking. Having clinical endpoints in addition to mortality will be a significant improvement to the literature. It has been suggested that effects of binge drinking could be an artifact due to misclassification or have a non-atherosclerotic pathology.[19;20]

The study will be able to look into more detail than has previously been possible on the long-term prognosis among patients with preexisting CVD or other high risk subgroups (RQ 1). In terms of drinking patterns, the results of individual level cohort studies mainly apply to cohorts who predominantly drink regularly with few heavy drinking occasions. Adequate power to investigate the combined effect of moderate and binge drinking requires large cohorts with enough data on the distribution of such drinking patterns within a population and information on exposure to other heart disease risk factors, pre-existing CVD as well as cognitive ability, mental distress and family data. [7]

We will in this study be able to investigate if the abstainers might be a selected group more closely, and for a wider range of potential underlying factors, than has previously been done (RQ 2 and 3). An assessment on how these factors relate to alcohol consumption and heart disease will also broaden our understanding on the clinical and public health impact of alcohol in groups at particularly high risk (RQ1 and 2).

The project will use novel approaches by taking advantage of family based designs to understand how both alcohol consumption pattern and CVD risk is patterned across generations and to study how much of the association between alcohol use and CVD may be accounted for by early life socioeconomic environment and shared family factors. Two approaches will be used: *i)* instrumental variable analysis using alcohol consumption among family members as instrument for own consumption and risk of CVD (RQ 4), and *ii)* fixed effect cotwin/sibling analysis (RQ 5) to adjust for shared early life socioeconomic environment and other family factors.

Alcohol consumption and cardiovascular risk may share common causes from early life and artificially inflate estimates of risk. Alcoholism is heritable (heritability 0.52-0.64)[22] and 50 % of the genes involved are shared by full siblings or parents.[23] Several studies suggest that parental alcohol use increases the risk of early alcohol debut and subsequent drinking patterns among children also indicating cultural heritability.[24;25] Our multigenerational approach investigate if alcohol use and CVD risk is jointly related across generations.

The association between moderate alcohol consumption and CVD mortality resembles the association between body mass index (BMI) and CVD mortality as both have a J-shaped relationship in which the lowest group (underweight and abstainers) has increased risk compared to those in the moderate categories.[26] Reverse causality by unmeasured health

problems may explain this pattern in these groups. In a Swedish intergenerational study, offspring BMI was used as an instrumental variable for own BMI as BMI in the offspring is less likely to be affected by health problems.[27] The authors found less adverse consequences of lower BMI compared to conventional observational studies. Alcohol consumption in family members can be used similarly as instrument for own consumption to avoid the problem of sick quitters potentially producing non-causal increased risk compared to moderate drinkers (RQ 4).[27] In both cases offspring alcohol and BMI are recorded earlier in life whereas CVD in parents is recorded at a higher age. More generally, using family proxies for exposure might be valuable not only to reduce reversed causality, but also because their alcohol intake will be less strongly associated with the individuals' other risk factors that predisposes him/her for CVD, i.e smoking and diet, and give less biased estimates of effect than own alcohol exposure.[28]

Data sources

The Cohort of Norway (CONOR) Data from10 epidemiological studies (n=181,891) from 1994-2003 have been merged into a national database.[29] All the surveys included self-reported health and selected diseases, various risk factors, socio-demographic factors, use of medications and physiological measures such as blood pressure, cholesterol, triglycerides, waist and hip circumference, height and weight.

The Norwegian Twin Registry (NTR) Panel II were born 1915-1960. Questionnaires on alcohol consumption were sent out in 1980 and 1990 and 14 500 responded to one of these. These questionnaires included detailed reports on health and risk factors.[30]

Norwegian Family Based Life course Study[31] Data from all 7,400 000 Norwegian citizens alive between 1960 to 2010 have been linked to family data and several health surveys and registers. Family linkage is available for more than 90 % of those born after 1940. This is linked to several other cardiovascular health surveys in addition to CONOR (in total 730,000) and the Conscript Registry.

Hospital discharges on cardiovascular diseases in Norway (FS-data): Data on all cardiovascular hospitalizations in Norway between 1994 and 2009 has been obtained through patient administrative systems at Norwegian hospitals. This includes coronary heart disease (both myocardial infarction and unstable angina), stroke and atrial fibrillation.

Samples (sub-populations)

RQ 1:

The CONOR and the NTR will be pooled together and the NTR will be used as a cohort for this purpose. This will include n=47,848 from CONOR and n=8028 individuals from NTR (n=55,876 in total) with complete data on both moderate and binge drinking. Mean year of birth is 1947 in CONOR and 1941 in the NTR sample. More deaths occur in the NTR (n=1453 deaths) compared to CONOR (n=1805 deaths). Number of CVD deaths in the CONOR sample with follow-up until 2010 is 547. Based on the proportion of CVD mortality and all-cause mortality and number of fatal and non-fatal cases in the FS-database, total number of cases, including both CONOR and the NTR is expected to be 3000 IHD and 3500 cases of stroke.

RQs 2 and 3:
This will include the CONOR and twin populations linked to the FS-data on hospital discharges, the Cause of Death Registry and the Conscript Registry. Two separate sub-populations of CONOR will be studied: all subjects who responded to items on moderate alcohol consumption (n=162,048) and, nested in this sample, those who responded to items on both moderate and binge drinking (n=47,848). In the first group there are 3774 deaths from CVD. There are in total 9735 acute myocardial infarction and 8926 stroke incident cases from the FS-database in CONOR. For the second group, see RQ1.

RQ 4:
This issue will be analyzed in a linkage between CONOR, cardiovascular health surveys in the Norwegian Family Based Life course study and the FS-data on hospital discharges. There are 116,037 individuals in CONOR with information on parents and siblings. Of these, 109,800 individuals have responded on the moderate alcohol consumption question with 9729 CVD deaths in fathers and 31,050 on the binge drinking question with 1854 CVD deaths occurring after the year of CONOR examination.

RQ 5:
CONOR: There are 48,401 siblings in CONOR with the same mother and father. 1025 of these have by 2010 died (281 CVD). We expect approximately 1400 CVD endpoints in the FS-database. For the second group on siblings, see RQ 1.

Questionnaire 1980 in NTR: In the NTR there are 5149 complete like-sexed twin pairs (n=10,298 individuals) who responded to the questionnaire on moderate alcohol consumption. 2021 individuals of these have died by 2010, including 810 CVD deaths. Based on the proportion CVD mortality and total mortality in the age group, number of fatal and non-fatal cases, and presuming they are followed up for hospital incidents from 1994, we expect approximately 1700 incident hospital cases of IHD and 1950 cases of cerebrovascular disease in the FS-database.

Questionnaire 1990 in NTR: And in the 1990 questionnaire there are 2138 complete twin pairs (n=4276 individuals) who have responded to the alcohol questionnaire in 1990 with information on both moderate and binge consumption. 626 of these individuals have died by 2010, 250 CVD deaths. Here we expect approximately 900 incident hospital cases of IHD and 1040 cases of cerebrovascular disease in the FS-database.

Main variables (see full description at FHI webpage)
Moderate alcohol consumption
Questions in CONOR:
1) “Approximately, how often have you during the last year consumed alcohol?” Eight response alternatives were recoded into five categories: Never/seldom, once per month, 2-3 per month, about once a week, 2-3 times a week, 4-7 times a week. 2) “When you drink, how many glasses do you usually drink?” 3) “When you drink, do you usually drink beer, wine or liquor?”
Questions in NTR:
1) “Do you drink beer?” 2) “Do you drink wine?” 3) “Do you drink liquor?” For each of these three questions, quantity was assessed by: “Do you drink less than a glass each day, 1-2 glasses each day or more than 2 glasses each day”.

Binge drinking

Questions in CONOR:

"Approximately how often during the past 12 months have you consumed alcohol corresponding to at least 5 glasses of liquor in 24 hours?"

In all 47,848 participants (26,151 men and 21,697 women), and answers varied from zero to 99 times during the last year. Nearly half (46.3%) reported zero, and the frequency was a higher in men than in women.

In NTR:

1990 questionnaire: "Approximately how many times during one year do you drink more than 5 bottles of bear, one bottle of red or white wine or a quarter bottle of liquor?" (Never, 1-4 times a year, 5-10 times a year, once a month, 2-3 times a month, once a week, 2-4 times a week or every day or almost every day?)

Life course socioeconomic position: Highest educational level, household income (yearly updated), Childhood parental occupational class and education, and housing conditions from Censuses 1960 onwards).

Cognitive ability/Intelligence in late adolescence: The Norwegian National Conscript Service examines more than 90% of the male population at age 18-19 years. General ability is a composite score based on 3 speeded subtests. The Arithmetic Test (25 minutes) has 30 items in prose and measures logical reasoning and arithmetic and algebraic ability; it is similar to the arithmetic test in the Wechsler Adult Intelligence Scale (WAIS). The Word Similarities Test (8 minutes) is a multiple-choice test with 54 items, akin to the vocabulary test in WAIS. The Figures Test (20 minutes) has 36 multiple-choice items with 6 or 8 alternatives and is similar to Raven's Progressive Matrices.[32]

The CONOR mental health index: This consists of summative index including seven questions on mental distress scored 1-4: "Have you, in the course of the last two weeks, felt: Nervous and unsettled? Troubled by anxiety? Secure and calm? Irritable? Happy and optimistic? Sad/depressed? Lonely?" Response categories are "No", "A little", "Quite a bit" and "Very". This index captures a combination of symptoms of anxiety and depression. This index has been validated, has good internal consistency and correlates highly with the total score for Hospital Anxiety and Depression Scale (HADS) (r=0.76) and with Hopkins symptom checklist (HSCL-10) (r=0.70).[33]

Cardiovascular risk factors: Current smoking, pack years of smoking, Body weight, Height, Waist circumference and Hip circumference, heart rate, systolic and diastolic blood pressures, non-fasting serum total, HDL cholesterol and triglycerides and physical activity.

Outcome variables:

Cause of death registry and CVD diagnoses from hospitals (1994-2009): circulatory (ICD-10: I00-I99), coronary heart disease (ICD-10: I20-I25), stroke (ICD-10: I60-I69). Cardiovascular Disease in Norway (CVDNOR) is a register that contains all hospital discharge diagnoses (obtained from Norwegian hospitals, FS-data) for cardiovascular disease and diabetes, and also relevant procedures, such as surgery and cardiac interventions. CVDNOR covers the whole Norwegian population 1994-2009.

Statistical methods

Sample size and power: The materials used to address RQ 1 is considerably higher powered than those used in previous research. Power calculations have been conducted using the sampsi command in STATA and the program Quanto for interaction terms. RQ 1: Two options have been used: Either to report the effect of moderate alcohol consumption adjusted for binge drinking or to test formally for interaction between average alcohol consumption and reported binge drinking. We have calculated the last because we consider this the most conservative approach. The relative risk of main effects has for average alcohol consumption reported to be 0.75 and for binge drinking 1.45.[9] If we assume that 10 % in the cohort develop CVD in the follow up period, power of 80 % and set p-value at 5 % for two sided tests, we would need a sample of 7,960 individuals, which is well below our sample of 55,876 individuals. RQ 2 and 3: Prevalence of mental distress in CONOR is on average 10 %, and the effect of depression on CVD is assumed to be 1.6. If we want to detect a 50 % difference in effect of binge between depressed and not depressed subjects, we would need 26,740 individuals (in our sample n=47,848), and for frequency of alcohol use 53,680 individuals (in our sample n=162,048). RQ 4: As we do not know the strength of association between alcohol consumption and sibling and parental CVD morbidity and mortality, it is difficult to calculate sample size exactly for this sub study. For this reason, we have preliminary investigated this issue, and found that it appears to be higher risk of CVD in fathers and same sexed sibling among those who are abstainers compared to those who drink moderately. The hazard ratio of CVD death in fathers among those who abstain from alcohol compared to moderate drinkers was 0.93 (0.88-0.99). RQ 5: It is not straightforward to estimate power in within sibling analyses. With a total of 5050 CVD endpoints, and based on our experiences in an analysis with similar design and number of endpoints[16], we consider it likely that we will be able to detect differences large enough to be theoretically interesting.

Data analysis: For time to event outcomes such as morbidity and mortality (RQ 1-4) the primary modelling strategy is to use Cox proportion hazard regression models. In RQ 1 the effect of moderate alcohol consumption will be analysed by adjusting for binge drinking and across strata of binge drinking (interaction term will be fitted to formally test if the effect differs). In RQ 2 and 3 the same strategy will be employed using strata of cognitive ability and mental health. In RQ 4 the aim is to see if using alcohol consumption among family members as instrumental variable will reproduce a protective effect of moderate alcohol consumption as seen in conventional analysis. To achieve this, z-scores will be calculated based on units of alcohol consumption reported. For RQ 5, the effects of alcohol consumption will be estimated by comparison between siblings or co-twins (“fixed effect”). This approach effectively matches out all factors shared between siblings that may confound the effect of alcohol on cardiovascular mortality reported in studies of unrelated individuals. The analyses will be conducted in the statistical software STATA. Age, smoking status and number of cigarettes per day in smokers, physical activity, self-reported disease (CVD, diabetes, antihypertensive medication) and psychosocial distress at baseline will be adjusted for as potential confounders.

The project plan, project management, organisation and cooperation

This project will be part of a larger collaboration at the Norwegian Institute of Public Health (NIPH) on alcohol and cardiovascular health which includes: Division of Epidemiology, Division of Toxicological and Pharmacological Assessment and Division of Mental Health. This group consists of the following researchers: Senior scientist Øyvind Næss (PI), Prof. Per Magnus (epidemiologist), Senior Scientist Sidsel Graff Iversen (epidemiologist), Post doc Inger Ariansen (epidemiologist), Scientist Gunn Peggy Knudsen (geneticist), Dr. Gudrun Høiseth (clinical pharmacologist), Prof. Jørg Mørland (clinical pharmacologist), Per Trygve Normann (clinical

pharmacologist), Senior Scientist Kristian Tambs (psychologist). This group has in a previous collaboration published a number of studies. [34-37]

The project will collaborate with the University of Bergen and the University of Bristol. Prof. Grethe Tell is PI for CVDNOR which provided the FS-data which includes cardiovascular hospital discharge data. Luisa Zuccolo is an epidemiologist working with George Davey Smith in Bristol on health consequences on alcohol consumption and was a visiting researcher at the NIPH in 2013. Øyvind Næss has previously collaborated with Davey Smith on other projects.

3. Key perspectives and compliance with strategic documents

The proposed study significantly enhances our understanding of the complex relationship between alcohol consumption and CVD. Our study will contribute with substantial new knowledge to this area of research. The information will be important in developing knowledge on the risk and benefit of alcohol consumption for CVD which will be important in developing future health policy on alcohol. The NIPH has CVD research as one of the key strategic areas of research and is currently strengthening this through the establishment of the Norwegian Heart Registry. Several divisions of the NIPH are working on alcohol related issues. NIPH is partly (with Wellcome Thrust) financing a large project (PI Dave Leon) on CVD in Russia where alcohol is an important component.

Ethics

The data linkage has been established and approved by the Regional Ethics Committee (REK Sørøst 11/1676) in the project "Socioeconomic inequalities in cardiovascular disease risk over the life course and in different generations" (Grant number 213788 Norwegian Research Council). An application will be sent to include the NTR and use the data linkage for the purpose of this project.

References

(1) World Health Organization. Global status report on alcohol and health. Geneva: 2011.

(2) Mackenbach JP. The persistene of health inequalities in modern welfare states: the explanation of a paradox. *Soc Sci Med* 2012; 75(4):761-9.

(3) Beck U. Risk Society. Towards a New Modernity. New Delhi: Sage:1992.

(4) Taylor B, Irving HM, Baliunas D, Roerecke M, Patra J, Mohapatra S et al. Alcohol and hypertension: gender differences in dose-response relationships determined through systematic review and meta-analysis. *Addiction* 2009; 104(12):1981-1990.

(5) Patra J, Taylor B, Irving H, Roerecke M, Baliunas D, Mohapatra S et al. Alcohol consumption and the risk of morbidity and mortality for different stroke types--a systematic review and meta-analysis. *BMC Public Health* 2010; 10:258.

(6) Samokhvalov AV, Irving HM, Rehm J. Alcohol consumption as a risk factor for atrial fibrillation: a systematic review and meta-analysis. *Eur J Cardiovasc Prev Rehabil* 2010; 17(6):706-712.

(7) Rehm J, Roerecke M. Alcohol, the heart and the cardiovascular system: what do we know and where should we go? *Drug Alcohol Rev* 2011; 30(4):335-337.

(8) Roerecke M, Rehm J. Alcohol intake revisited: risks and benefits. *Curr Atheroscler Rep* 2012; 14(6):556-562.

(9) Roerecke M, Rehm J. Irregular heavy drinking occasions and risk of ischemic heart disease: a systematic review and meta-analysis. *Am J Epidemiol* 2010; 171(6):633-644.

(10) Andreasson S. The health benefits of moderate alcohol consumption called into question. *Addiction Research Theory* 2007; 15:3-5.

(11) Pedersen W. Avholdende - en risikogruppe? *Tidsskr Nor Laegeforen* 2013; 133:33-36.

(12) Corley J, Jia X, Brett CE, Gow AJ, Starr JM, Kyle JA et al. Alcohol intake and cognitive abilities in old age: the Lothian Birth Cohort 1936 study. *Neuropsychology* 2011; 25(2):166-175.

(13) Muller M, Kowalewski R, Metzler S, Stettbacher A, Rossler W, Vetter S. Associations between IQ and alcohol consumption in a population of young males: a large database analysis. *Soc Psychiatry Psychiatr Epidemiol* 2013.

(14) Fillmore KM, Stockwell T, Chikritzhs T, Bostrom A, Kerr W. Moderate alcohol use and reduced mortality risk: systematic error in prospective studies and new hypotheses. *Ann Epidemiol* 2007; 17(5 Suppl):S16-S23.

(15) Heim D. Editorial: Contested knowledge: Introducing Fillmore, Kerr, Stockwell, Chikritzhs, and bostrom (2006). *Addiction Research Theory* 2006; 14:97-99.

(16) Naess O, Hoff DA, Lawlor D, Mortensen LH. Education and adult cause-specific mortality--examining the impact of family factors shared by 871 367 Norwegian siblings. *Int J Epidemiol* 2012; 41(6):1683-1691.

(17) Horverak Ø, Bye E K. The Norwegian drinking pattern. A study based on analyses of survey data 1973-2004. Oslo: Norwegian Institute of Alcohol and Drug Research; 2007.

(18) Smith GD. Epidemiology, epigenetics and the 'Gloomy Prospect': embracing randomness in population health research and practice. *Int J Epidemiol* 2011; 40(3):537-562.

(19) Leon DA, Shkolnikov VM, Mckee M, Kiryanov N, Andreev E. Alcohol increases circulatory disease mortality in Russia: acute and chronic effects or misattribution of cause? *Int J Epidemiol* 2010; 39(5):1279-1290.

(20) Sidorenkov O, Nilssen O, Nieboer E, Kleshchinov N, Grjibovski AM. Premature cardiovascular mortality and alcohol consumption before death in Arkhangelsk, Russia: an analysis of a consecutive series of forensic autopsies. *Int J Epidemiol* 2011; 40(6):1519-1529.

(21) Graff-Iversen S, Jansen MD, Hoff DA, Hoiseth G, Knudsen GP, Magnus P et al. Divergent associations of drinking frequency and binge consumption of alcohol with mortality within the same cohort. *J Epidemiol Community Health* 2013; 67(4):350-357.

(22) Enoch MA. Genetic and environmental influences on the development of alcoholism: resilience vs. risk. *Ann N Y Acad Sci* 2006; 1094:193-201.

(23) Lawlor DA, Mishra GD. Family Matters. Designing, analysing and understanding family-based studies in life-course epidemiology. New York: Oxford University Press; 2009.

(24) Seljamo S, Aromaa M, Koivusilta L, Rautava P, Sourander A, Helenius H et al. Alcohol use in families: a 15-year prospective follow-up study. *Addiction* 2006; 101(7):984-992.

(25) White HR, Johnson V, Buyske S. Parental modeling and parenting behavior effects on offspring alcohol and cigarette use. A growth curve analysis. *J Subst Abuse* 2000; 12(3):287-310.

(26) Davey SG, Sterne JA, Fraser A, Tynelius P, Lawlor DA, Rasmussen F. The association between BMI and mortality using offspring BMI as an indicator of own BMI: large intergenerational mortality study. *BMJ* 2009; 339:b5043.

(27) Greenland S. An introduction to instrumental variables for epidemiologists. *International Journal of Epidemiology* 2000; 29(4):722-729.

(28) Smith GD, Lawlor DA, Harbord R, Timpson N, Day I, Ebrahim S. Clustered environments and randomized genes: a fundamental distinction between conventional and genetic epidemiology. *PLoS Med* 2007; 4(12):e352.

(29) Naess O, Sogaard AJ, Arnesen E, Beckstrom AC, Bjertness E, Engeland A et al. Cohort Profile: Cohort of Norway. *Int J Epidemiol* 2008.

(30) Nilsen TS, Knudsen GP, Gervin K, Brandt I, Roysamb E, Tambs K et al. The Norwegian Twin Registry from a public health perspective: a research update. *Twin Res Hum Genet* 2013; 16(1):285-295.

(31) Naess O, Hoff DA. The Norwegian Family Based Life Course (NFLC) study: data structure and potential for public health research. *Int J Public Health* 2013; 58(1):57-64.

(32) Eriksen W, Sundet JM, Tambs K. Birth weight standardized to gestational age and intelligence in young adulthood: a register-based birth cohort study of male siblings. *Am J Epidemiol* 2010; 172(5):530-536.

(33) Søgaard AJ, Bjelland I, Tell GS, Røysamb E. A comparison of the CONOR Mental Health Index to the HSCL-10 and HADS. *Norsk Epidemiologi* 2003; 13:279-284.

(34) Dverdal M, Tambs, Høiseth G, Mørland J, Myhre R, Næss Ø et al. Genetic variants in loci 1p13 and 9p21 and fatal coronary heart disease in a Norwegian case-cohort study. *Molecular Biology Reports* (in press) 2014.

(35) Graff-Iversen S, Jansen MD, Hoff DA, Hoiseth G, Knudsen GP, Magnus P et al. Divergent associations of drinking frequency and binge consumption of alcohol with mortality within the same cohort. *J Epidemiol Community Health* 2013; 67(4):350-357.

(36) Hoiseth G, Magnus P, Knudsen GP, Jansen MD, Naess O, Tambs K et al. Is ADH1C genotype relevant for the cardioprotective effect of alcohol? *Alcohol* 2013; 47(2):81-84.

(37) Magnus P, Bakke E, Hoff DA, Hoiseth G, Graff-Iversen S, Knudsen GP et al. Controlling for high-density lipoprotein cholesterol does not affect the magnitude of the relationship between alcohol and coronary heart disease. *Circulation* 2011; 124(21):2296-2302.
