# Supplementary material for: Association of coincident self-reported mental health problems and alcohol intake with all-cause and cardiovascular disease mortality: A Norwegian pooled population analysis
Source: PLoS Med. 2020 Feb 3;17(2):e1003030. doi: 10.1371/journal.pmed.1003030 (PMC6996806; doi:10.1371/journal.pmed.1003030)
Supplement: S1 Table — (DOCX) [file pmed.1003030.s005.docx]

|  | | **Survey year  (range)** | **Response rate (%)** | **Participants  (n)** | **Age  (mean ± SD)** | **Age  (range)** | **Sex  (male %)** | **Alcohol  data** |
| --- | --- | --- | --- | --- | --- | --- | --- | --- |
| **Age 40 program** | |  |  |  |  |  |  |  |
|  | **Wave 3** | 1994-1997 | 66.7 | 73,781 | 42.1 ± 5.3 | (15 – 76) | 47.4 | A |
|  | **Wave 4** | 1997-1999 | 61.0 | 66,303 | 40.9 ± 1.2 | (36 – 45) | 46.3 | A |
| **Cohort of Norway (CONOR)** | |  |  |  |  |  |  |  |
|  | **HUBRO** | 1996-2001 | 37.5 | 21,220 | 48.4 ± 15.5 | (29.9 – 77.7) | 44.5 | B |
|  | **OPPHED** | 2000-2001 | 55.5 | 12,196 | 50.1 ± 14.3 | (29.9 – 76.8) | 45.8 | B |
|  | **TROMSØ 4** | 1994-1995 | 71.7 | 26,902 | 47.5 ± 15.1 | (24.7 – 97.2) | 44.3 | A |
|  | **TROMSØ 5** | 2001 | 78.0 | 7880 | 36.9 ± 10.7 | (29.5 – 80.9) | 47.5 | B |
|  | **HUSK** | 1997-1999 | 66.2 | 25,464 | 47.7 ± 9.8 | (39.8 – 74.0) | 45.8 | A |
|  | **TROFINN** | 2002 | 57.5 | 8869 | 56.5 ± 12.7 | (29.0 – 77.7) | 46.5 | B |
|  | **HUNT 2** | 1995-1997 | 69.0 | 64,926 | 50.3 ± 17.4 | (18 – 103.4) | 46.8 | A |

Questions used to assess the amount of alcohol consumed on average per day:

1. “How many glasses of beer, wine, or spirits do you usually drink during a two-week period?”
2. “How often during the past 12 months have you consumed alcohol?” in combination with the question “When you drank alcohol, how many glasses did you usually drink?”.
