# Supplementary material for: Association of coincident self-reported mental health problems and alcohol intake with all-cause and cardiovascular disease mortality: A Norwegian pooled population analysis
Source: PLoS Med. 2020 Feb 3;17(2):e1003030. doi: 10.1371/journal.pmed.1003030 (PMC6996806; doi:10.1371/journal.pmed.1003030)
Supplement: S2 Table — (DOCX) [file pmed.1003030.s006.docx]

|  | **Source population** |  | **Excluded for missing values** | | | | | | |  | **Study population** |
| --- | --- | --- | --- | --- | --- | --- | --- | --- | --- | --- | --- |
| **Variable** | **Individual  participants**  **(n = 294,992*)** |  | **Drinking  status**  **(n=6521*)** |  | **Mental health  index**  **(n=28,278)** |  | **Average intake  of alcohol (among  current drinkers) (n=8713)** |  | **Other  covariates**  **(n=8087)** |  | **Complete  cases  (n=243,372)** |
| Age | 45.9 (12.2) |  | 57.4 (17.1) |  | 53.0 (15.4) |  | 50.3 (15.5) |  | 52.8 (16.4) |  | 43.9 (10.6) |
| Sex (male), n (%) | 137,518 (46.6) |  | 2724 (41.8) |  | 11,693 (41.4) |  | 3323 (38.1) |  | 3553 (43.9) |  | 116,218 (47.8) |
| Education (1-8) | 3.9 (1.6) |  | 3.1 (1.4) |  | 3.3 (1.5) |  | 3.4 (1.5) |  | 3.4 (1.6) |  | 4.1 (1.6) |
| Birth year | 1951 (12.2) |  | 1940 (17.5) |  | 1945 (15.9) |  | 1948 (15.2) |  | 1944 (16.8) |  | 1952 (10.7) |
| Deaths, n (%) | 34,680 (11.8) |  | 2391 (36.7) |  | 7027 (24.8) |  | 1647 (18.9) |  | 2233 (27.6) |  | 21,376 (8.8) |
| CVD deaths, n (%) | 11,727 (4.0) |  | 993 (15.2) |  | 2690 (9.5) |  | 582 (6.7) |  | 870 (10.8) |  | 6587 (2.7) |

*Individuals (n = 134) with missing data on drinking status in a survey in the Cohort of Norway and data on drinking status from a survey in the Age 40 Program were not described here. Age, education and birth year presented as mean (standard deviation). Abbreviations: CVD, cardiovascular disease.
