# Supplementary material for: Association of coincident self-reported mental health problems and alcohol intake with all-cause and cardiovascular disease mortality: A Norwegian pooled population analysis
Source: PLoS Med. 2020 Feb 3;17(2):e1003030. doi: 10.1371/journal.pmed.1003030 (PMC6996806; doi:10.1371/journal.pmed.1003030)
Supplement: S3 Table — (DOCX) [file pmed.1003030.s007.docx]

|  |  |  |  |  | **Average intake of alcohol (grams per day)** | | | |  |
| --- | --- | --- | --- | --- | --- | --- | --- | --- | --- |
| **Characteristics** | **Mental  Health Index  (mean score)** | ***n*** | **All**  **participants**  **(*n* = 243,372)** | **Current  abstainers**  **(*n* = 22,496)** | **Low**  **<2 g/day**  **(*n* = 85,961)** | **Light**  **2-11.99 g/day**  **(*n* = 116,170)** | **Moderate**  **12-23.99 g/day**  **(*n* = 15,944)** | **High**  **≥24 g/day (*n* = 2801)** | ***p-value*** |
| Never smoker | All | 243,372 | 95,248 (39.1 %) | 14,443 (64.2 %) | 35,257 (41.0 %) | 40,681 (35.0 %) | 4265 (26.7 %) | 602 (21.5 %) | <0.001 |
|  | 1.00 – 1.50 | 148,428 | 61,543 (41.5 %) | 8969 (68.1 %) | 22,656 (43.2 %) | 26,917 (37.2 %) | 2660 (29.1 %) | 341 (25.3 %) | <0.001 |
|  | 1.51 – 2.00 | 71,546 | 26,577 (37.1 %) | 4063 (62.9 %) | 9826 (39.4 %) | 11,232 (32.9 %) | 1274 (25.3 %) | 182 (19.6 %) | <0.001 |
|  | 2.01 – 4.00 | 23,398 | 7128 (30.5 %) | 1411 (49.2 %) | 2775 (32.4 %) | 2532 (26.1 %) | 331 (18.9 %) | 79 (15.0 %) | <0.001 |
|  | *p-value* |  | <0.001 | <0.001 | <0.001 | <0.001 | <0.001 | <0.001 |  |
| Former light smoker | All | 243,372 | 57,783 (23.7 %) | 3979 (17.7 %) | 20,043 (23.3 %) | 29,448 (25.3 %) | 3747 (23.5 %) | 566 (20.2 %) | <0.001 |
|  | 1.00 – 1.50 | 148,428 | 35,819 (24.1 %) | 2295 (17.4 %) | 12,305 (23.5 %) | 18,588 (25.7 %) | 2336 (25.5 %) | 295 (21.9 %) | <0.001 |
|  | 1.51 – 2.00 | 71,546 | 17,303 (24.2 %) | 1191 (18.5 %) | 5951 (23.8 %) | 8859 (25.9 %) | 1095 (21.7 %) | 207 (22.3 %) | <0.001 |
|  | 2.01 – 4.00 | 23,398 | 4661 (19.9 %) | 493 (17.2 %) | 1787 (20.9 %) | 2001 (20.7 %) | 316 (18.0 %) | 64 (12.2 %) | <0.001 |
|  | *p-value* |  | <0.001 | 0.157 | <0.001 | <0.001 | <0.001 |  |  |
| Former heavy smoker | All | 243,372 | 6982 (2.9 %) | 439 (2.0 %) | 2236 (2.6 %) | 3343 (2.9 %) | 779 (4.9 %) | 185 (6.6 %) | <0.001 |
|  | 1.00 – 1.50 | 148,428 | 3954 (2.7 %) | 214 (1.6 %) | 1262 (2.4 %) | 1956 (2.7 %) | 422 (4.6 %) | 100 (7.4 %) | <0.001 |
|  | 1.51 – 2.00 | 71,546 | 2240 (3.1 %) | 131 (2.0 %) | 706 (2.8 %) | 1059 (3.1 %) | 281 (5.6 %) | 63 (6.8 %) | <0.001 |
|  | 2.01 – 4.00 | 23,398 | 788 (3.4 %) | 94 (3.3 %) | 268 (3.1 %) | 328 (3.4 %) | 76 (4.3 %) | 22 (4.2 %) | 0.102 |
|  | *p-value* |  | <0.001 | <0.001 | <0.001 | <0.001 | <0.001 | 0.039 |  |
| Current light smoker | All | 243,372 | 64,499 (26.5 %) | 2664 (11.8 %) | 22,834 (26.6 %) | 33,545 (28.9 %) | 4714 (29.6 %) | 742 (26.5 %) | <0.001 |
|  | 1.00 – 1.50 | 148,428 | 37,332 (25.2 %) | 1299 (9.9 %) | 13,311 (25.4 %) | 19,881 (27.5 %) | 2519 (27.5 %) | 322 (23.9 %) | <0.001 |
|  | 1.51 – 2.00 | 71,546 | 19,557 (27.3 %) | 799 (12.4 %) | 6750 (27.0 %) | 10,170 (29.8 %) | 1575 (31.3 %) | 263 (28.3 %) | <0.001 |
|  | 2.01 – 4.00 | 23,398 | 7610 (32.5 %) | 566 (19.7 %) | 2773 (32.4 %) | 3494 (36.1 %) | 620 (35.4 %) | 157 (29.9 %) | <0.001 |
|  | *p-value* |  | <0.001 | <0.001 | <0.001 | <0.001 | <0.001 | 0.009 |  |
| Current heavy smoker | All | 243,372 | 18,860 (7.7 %) | 971 (4.3 %) | 5591 (6.5 %) | 9153 (7.9 %) | 2439 (15.3 %) | 706 (25.2 %) | <0.001 |
|  | 1.00 – 1.50 | 148,428 | 9780 (6.6 %) | 397 (3.0 %) | 2905 (5.5 %) | 4973 (6.9 %) | 1216 (13.3 %) | 289 (21.5 %) | <0.001 |
|  | 1.51 – 2.00 | 71,546 | 5869 (8.2 %) | 271 (4.2 %) | 1723 (6.9 %) | 2848 (8.3 %) | 813 (16.1 %) | 214 (23.0 %) | <0.001 |
|  | 2.01 – 4.00 | 23,398 | 3211 (13.7 %) | 303 (10.6 %) | 963 (11.2 %) | 1332 (13.8 %) | 410 (23.4 %) | 203 (38.7 %) | <0.001 |
|  | *p-value* |  | <0.001 | <0.001 | <0.001 | <0.001 | <0.001 | <0.001 |  |
| Heart rate (bpm) | All | 243,372 | 73.0 (12.2) | 74.5 (12.9) | 74.0 (12.3) | 72.1 (12.0) | 71.9 (12.1) | 73.5 (12.7) | <0.001 |
|  | 1.00 – 1.50 | 148,428 | 72.6 (12.1) | 74.0 (12.6) | 73.6 (12.2) | 71.8 (11.9) | 71.2 (11.9) | 72.3 (12.7) | <0.001 |
|  | 1.51 – 2.00 | 71,546 | 73.1 (12.2) | 74.6 (12.9) | 74.2 (12.3) | 72.2 (11.9) | 72.2 (12.2) | 73.2 (12.2) | <0.001 |
|  | 2.01 – 4.00 | 23,398 | 74.9 (12.6) | 76.7 (13.7) | 75.4 (12.5) | 73.9 (12.3) | 74.3 (13.1) | 77.2 (13.1) | <0.001 |
|  | *p-value* |  | <0.001 | <0.001 | <0.001 | <0.001 | <0.001 | <0.001 |  |
| Triglycerides (mmol/l) | All | 243,372 | 1.70(1.17) | 1.76 (1.15) | 1.67 (1.13) | 1.67 (1.15) | 1.88 (1.37) | 2.00 (1.52) | <0.001 |
|  | 1.00 – 1.50 | 148,428 | 1.69 (1.14) | 1.72 (1.11) | 1.67 (1.11) | 1.67 (1.14) | 1.87 (1.34) | 1.94 (1.32) | <0.001 |
|  | 1.51 – 2.00 | 71,546 | 1.69 (1.19) | 1.77 (1.18) | 1.67 (1.17) | 1.66 (1.14) | 1.89 (1.40) | 2.07 (1.67) | <0.001 |
|  | 2.01 – 4.00 | 23,398 | 1.75 (1.24) | 1.89 (1.28) | 1.71 (1.16) | 1.70 (1.22) | 1.92 (1.44) | 2.03 (1.72) | 0.571 |
|  | *p-value* |  | <0.001 | <0.001 | 0.013 | 0.581 | 0.219 | 0.119 |  |
|  |  |  |  |  |  |  |  |  |  |
| Total cholesterol (mmol/l) | All | 243,372 | 5.66 (1.13) | 5.83 (1.38) | 5.68 (1.14) | 5.60 (1.07) | 5.74 (1.13) | 5.81 (1.11) | <0.001 |
|  | 1.00 – 1.50 | 148,428 | 5.67 (1.13) | 5.83 (1.47) | 5.68 (1.14) | 5.61 (1.06) | 5.76 (1.07) | 5.83 (1.10) | <0.001 |
|  | 1.51 – 2.00 | 71,546 | 5.64 (1.12) | 5.80 (1.24) | 5.66 (1.14) | 5.58 (1.07) | 5.73 (1.22) | 5.81 (1.07) | <0.001 |
|  | 2.01 – 4.00 | 23,398 | 5.69 (1.15) | 5.90 (1.28) | 5.71 (1.16) | 5.61 (1.09) | 5.72 (1.15) | 5.76 (1.23) | <0.001 |
|  | *p-value* |  | 0.816 | <0.001 | 0.593 | 0.019 | 0.054 | 0.212 |  |
| HDL–C (mmol/l), women | All | 118,688 | 1.50 (0.37) | 1.44 (0.37) | 1.46 (0.36) | 1.54 (0.37) | 1.65 (0.41) | 1.73 (0.47) | <0.001 |
|  | 1.00 – 1.50 | 69,455 | 1.51 (0.37) | 1.46 (0.37) | 1.47 (0.36) | 1.55 (0.37) | 1.67 (0.42) | 1.72 (0.46) | <0.001 |
|  | 1.51 – 2.00 | 36,258 | 1.49 (0.37) | 1.43 (0.36) | 1.45 (0.36) | 1.54 (0.37) | 1.63 (0.40) | 1.73 (0.50) | <0.001 |
|  | 2.01 – 4.00 | 12,975 | 1.47 (0.39) | 1.4 (0.38) | 1.42 (0.37) | 1.52 (0.39) | 1.63 (0.39) | 1.72 (0.42) | <0.001 |
|  | *p-value* |  | <0.001 | <0.001 | <0.001 | <0.001 | 0.018 | 0.941 |  |
| HDL–C (mmol/l), men | All | 108,125 | 1.23 (0.33) | 1.17 (0.31) | 1.19 (0.31) | 1.25 (0.32) | 1.30 (0.35) | 1.38 (0.41) | <0.001 |
|  | 1.00 – 1.50 | 68,663 | 1.23 (0.32) | 1.17 (0.30) | 1.19 (0.31) | 1.25 (0.32) | 1.30 (0.34) | 1.38 (0.39) | <0.001 |
|  | 1.51 – 2.00 | 30,529 | 1.24 (0.33) | 1.17 (0.32) | 1.19 (0.32) | 1.25 (0.32) | 1.30 (0.35) | 1.36 (0.40) | <0.001 |
|  | 2.01 – 4.00 | 8933 | 1.22 (0.33) | 1.13 (0.3) | 1.17 (0.31) | 1.23 (0.32) | 1.29 (0.35) | 1.41 (0.47) | <0.00 |
|  | *p-value* |  | 0.019 | 0.344 | 0.002 | 0.050 | 0.285 | 0.524 |  |
| Diabetes | All | 243,372 | 3502 (1.4 %) | 778 (3.5 %) | 1460 (1.7 %) | 1107 (1.0 %) | 119 (0.7 %) | 38 (1.4 %) | <0.001 |
|  | 1.00 – 1.50 | 148,428 | 1882 (1.3 %) | 380 (2.9 %) | 781 (1.5 %) | 637 (0.9 %) | 68 (0.7 %) | 16 (1.2 %) | <0.001 |
|  | 1.51 – 2.00 | 71,546 | 1096 (1.5 %) | 240 (3.7 %) | 473 (1.9 %) | 342 (1.0 %) | 30 (0.6 %) | 11 (1.2 %) | <0.001 |
|  | 2.01 – 4.00 | 23,398 | 524 (2.2 %) | 158 (5.5 %) | 206 (2.4 %) | 128 (1.3 %) | 21 (1.2 %) | 11 (2.1 %) | <0.001 |
|  | *p-value* |  | <0.001 | <0.001 | <0.001 | <0.001 | 0.041 | 0.268 |  |
| History of CVD | All | 243,372 | 7424 (3.1 %) | 1504 (6.7 %) | 3063 (3.6 %) | 2396 (2.1 %) | 364 (2.3 %) | 97 (3.5 %) | <0.001 |
|  | 1.00 – 1.50 | 148,428 | 3971 (2.7 %) | 753 (5.7 %) | 1665 (3.2 %) | 1340 (1.9 %) | 180 (2.0 %) | 33 (2.4 %) | <0.001 |
|  | 1.51 – 2.00 | 71,546 | 2330 (3.3 %) | 476 (7.4 %) | 965 (3.9 %) | 730 (2.1 %) | 131 (2.6 %) | 28 (3.0 %) | <0.001 |
|  | 2.01 – 4.00 | 23,398 | 1123 (4.8 %) | 275 (9.6 %) | 433 (5.1 %) | 326 (3.4 %) | 53 (3.0 %) | 36 (6.9 %) | <0.001 |
|  | *p-value* |  | <0.001 | <0.001 | <0.001 | <0.001 | 0.005 | <0.001 |  |
| Family history of coronary heart disease | All | 243,372 | 97,469 (40.0 %) | 9528 (42.4 %) | 35,212 (41.0 %) | 45,653 (39.3 %) | 6004 (37.7 %) | 1072 (38.3 %) | <0.001 |
|  | 1.00 – 1.50 | 148,428 | 57,896 (39.0 %) | 5447 (41.3 %) | 20,844 (39.7 %) | 27,786 (38.4 %) | 3319 (36.3 %) | 500 (37.1 %) | <0.001 |
|  | 1.51 – 2.00 | 71,546 | 29,358 (41.0 %) | 2786 (43.2 %) | 10,528 (42.2 %) | 13,725 (40.2 %) | 1966 (39.0 %) | 353 (38.0 %) | <0.001 |
|  | 2.01 – 4.00 | 23,398 | 10,215 (43.7 %) | 1295 (45.2 %) | 3840 (44.8 %) | 4142 (42.8 %) | 719 (41.0 %) | 219 (41.7 %) | 0.003 |
|  | *p-value* |  | <0.001 | <0.001 | <0.001 | <0.001 | <0.001 | 0.181 |  |

Values are presented as mean (standard deviation) or count (percentage). Group differences were assessed with analysis of variance and the chi-squared test. Mental health index is presented as the mean score per question (range 1 – 4) on the 7 questions that constitutes the mental health index, where a high score indicates a greater number of self-reported mental health issues. Abbreviations: HDL-C, high density lipoprotein cholesterol; CVD, cardiovascular disease.
