# Supplementary material for: Association of coincident self-reported mental health problems and alcohol intake with all-cause and cardiovascular disease mortality: A Norwegian pooled population analysis
Source: PLoS Med. 2020 Feb 3;17(2):e1003030. doi: 10.1371/journal.pmed.1003030 (PMC6996806; doi:10.1371/journal.pmed.1003030)
Supplement: S4 Table — (DOCX) [file pmed.1003030.s008.docx]

| **HR (95 % CI) in the  sample and in strata of alcohol intake** | | **Mental health problems (mean score per question on the mental health index, range 1-4)** | | | | | |
| --- | --- | --- | --- | --- | --- | --- | --- |
|  |  | **1.00 – 1.50**  (*n* = 148,428) | **1.51 – 2.00**  (*n* = 71,546) | **2.01 – 4.00** (*n* = 23,398) |  | **Per mean score increase**  (*n* = 220,876) | **Difference in slope (interaction term)** |
| *All-cause mortality* | |  |  |  |  |  |  |
| Unadjusted | |  |  |  |  |  |  |
|  | Overall | 1.00 | 1.07 (1.04, 1.10), p < 0.001 | 1.55 (1.48, 1.61), p < 0.001 |  | 1.35 (1.32, 1.39), p < 0.001 | - |
|  | Current abstainer | 1.00 | 1.17 (1.09, 1.25), p < 0.001 | 1.47 (1.35, 1.60), p < 0.001 |  | 1.30 (1.23, 1.38), p < 0.001 | 1.06 (0.98, 1.14), p = 0.146 |
|  | <2 g/day | 1.00 | 1.08 (1.03, 1.13), p = 0.001 | 1.35 (1.26, 1.44), p < 0.001 |  | 1.23 (1.18, 1.29), p < 0.001 | Referent |
|  | 2 – 11.99 g/day | 1.00 | 0.99 (0.94, 1.04), p = 0.715 | 1.54 (1.43, 1.66), p < 0.001 |  | 1.32 (1.25, 1.39), p < 0.001 | 1.07 (0.99, 1.15), p = 0.069 |
|  | 12 – 23.99 g/day | 1.00 | 1.04 (0.92, 1.19), p = 0.523 | 1.54 (1.31, 1.82), p < 0.001 |  | 1.47 (1.31, 1.66), p < 0.001 | 1.19 (1.05, 1.35), p = 0.006 |
|  | ≥24 g/day | 1.00 | 1.22 (0.95, 1.56), p = 0.113 | 1.86 (1.43, 2.40), p < 0.001 |  | 1.53 (1.30, 1.80), p < 0.001 | 1.24 (1.05, 1.47), p = 0.012 |
|  |  |  |  |  |  |  |  |
| + age and sex | |  |  |  |  |  |  |
|  | Overall | 1.00 | 1.10 (1.06, 1.13), p < 0.001 | 1.56 (1.50, 1.62), p < 0.001 |  | 1.38 (1.34, 1.42), p < 0.001 | - |
|  | Current abstainer | 1.00 | 1.15 (1.07, 1.23), p < 0.001 | 1.51 (1.38, 1.64), p < 0.001 |  | 1.33 (1.26, 1.41), p < 0.001 | 1.04 (0.97, 1.12), p = 0.293 |
|  | <2 g/day | 1.00 | 1.10 (1.05, 1.15), p < 0.001 | 1.43 (1.33, 1.53), p < 0.001 |  | 1.28 (1.23, 1.35), p < 0.001 | Referent |
|  | 2 – 11.99 g/day | 1.00 | 1.04 (0.99, 1.10), p = 0.117 | 1.60 (1.48, 1.72), p < 0.001 |  | 1.41 (1.33, 1.49), p < 0.001 | 1.10 (1.03, 1.19), p = 0.007 |
|  | 12 – 23.99 g/day | 1.00 | 1.14 (1.00, 1.30), p = 0.059 | 1.77 (1.50, 2.09), p < 0.001 |  | 1.63 (1.46, 1.84), p < 0.001 | 1.30 (1.15, 1.48), p < 0.001 |
|  | ≥24 g/day | 1.00 | 1.29 (1.01, 1.65), p = 0.047 | 2.67 (2.05, 3.48), p < 0.001 |  | 1.91 (1.62, 2.25), p < 0.001 | 1.63 (1.38, 1.93), p < 0.001 |
|  |  |  |  |  |  |  |  |
| + multivariable | |  |  |  |  |  |  |
|  | Overall | 1.00 | 1.03 (1.00, 1.06), p = 0.072 | 1.26 (1.21, 1.32), p < 0.001 |  | 1.17 (1.14, 1.21), p < 0.001 | - |
|  | Current abstainer | 1.00 | 1.06 (0.99, 1.13), p = 0.110 | 1.16 (1.06, 1.27), p = 0.001 |  | 1.10 (1.03, 1.17), p = 0.002 | 0.98 (0.90, 1.05), p = 0.511 |
|  | <2 g/day | 1.00 | 1.04 (0.99, 1.09), p = 0.104 | 1.25 (1.17, 1.34), p < 0.001 |  | 1.15 (1.10, 1.21), p < 0.001 | Referent |
|  | 2 – 11.99 g/day | 1.00 | 0.99 (0.94, 1.05), p = 0.756 | 1.31 (1.22, 1.42), p < 0.001 |  | 1.20 (1.14, 1.27), p < 0.001 | 1.07 (1.00, 1.15), *p* = 0.057 |
|  | 12 – 23.99 g/day | 1.00 | 1.01 (0.89, 1.15), p = 0.861 | 1.32 (1.11, 1.57), p = 0.001 |  | 1.32 (1.17, 1.48), p < 0.001 | 1.22 (1.08, 1.38), *p* = 0.002 |
|  | ≥24 g/day | 1.00 | 1.14 (0.88, 1.46), p = 0.341 | 1.59 (1.19, 2.12), p = 0.002 |  | 1.32 (1.11, 1.58), p = 0.002 | 1.38 (1.16, 1.63), *p* < 0.001 |
|  | |  |  |  |  |  |  |
| *CVD mortality* | |  |  |  |  |  |  |
| Unadjusted | |  |  |  |  |  |  |
|  | Overall | 1.00 | 1.07 (1.01, 1.13), p = 0.022 | 1.38 (1.28, 1.49), p < 0.001 |  | 1.26 (1.19, 1.32), p < 0.001 | - |
|  | Current abstainer | 1.00 | 1.16 (1.04, 1.29), p = 0.009 | 1.39 (1.21, 1.60), p < 0.001 |  | 1.26 (1.15, 1.38), p < 0.001 | 1.08 (0.95, 1.23), p = 0.228 |
|  | <2 g/day | 1.00 | 1.09 (1.00, 1.19), p = 0.045 | 1.21 (1.08, 1.37), p = 0.002 |  | 1.17 (1.07, 1.27), p < 0.001 | Referent |
|  | 2 – 11.99 g/day | 1.00 | 0.94 (0.85, 1.05), p = 0.266 | 1.24 (1.07, 1.45), p = 0.006 |  | 1.13 (1.01, 1.27), p = 0.027 | 0.97 (0.84, 1.12), p = 0.670 |
|  | 12 – 23.99 g/day | 1.00 | 1.15 (0.89, 1.49), p = 0.276 | 1.13 (0.78, 1.66), p = 0.515 |  | 1.18 (0.91, 1.52), p = 0.219 | 1.01 (0.77, 1.32), p = 0.971 |
|  | ≥24 g/day | 1.00 | 1.09 (0.65, 1.82), p = 0.753 | 1.58 (0.91, 2.75), p = 0.103 |  | 1.40 (0.98, 2.00), p = 0.061 | 1.21 (0.84, 1.74), p = 0.306 |
|  |  |  |  |  |  |  |  |
| + age and sex | |  |  |  |  |  |  |
|  | Overall | 1.00 | 1.10 (1.04, 1.16), p = 0.001 | 1.39 (1.29, 1.50), p < 0.001 |  | 1.29 (1.22, 1.36), p < 0.001 | - |
|  | Current abstainer | 1.00 | 1.12 (1.00, 1.25), p = 0.035 | 1.39 (1.21, 1.60), p < 0.001 |  | 1.27 (1.15, 1.40), p < 0.001 | 1.05 (0.92, 1.19), p = 0.446 |
|  | <2 g/day | 1.00 | 1.12 (1.03, 1.21), p = 0.012 | 1.31 (1.16, 1.48), p < 0.001 |  | 1.23 (1.13, 1.34), p < 0.001 | Referent |
|  | 2 – 11.99 g/day | 1.00 | 1.02 (0.92, 1.13), p = 0.751 | 1.33 (1.14, 1.55), p < 0.001 |  | 1.26 (1.12, 1.41), p < 0.001 | 1.01 (0.88, 1.16), p = 0.854 |
|  | 12 – 23.99 g/day | 1.00 | 1.30 (1.00, 1.67), p = 0.046 | 1.44 (0.98, 2.11), p = 0.062 |  | 1.42 (1.10, 1.83), p = 0.007 | 1.16 (0.89, 1.52), p = 0.266 |
|  | ≥24 g/day | 1.00 | 1.17 (0.70, 1.96),p = 0.575 | 2.59 (1.47, 4.57), p = 0.001 |  | 1.90 (1.33, 2.71), p < 0.001 | 1.74 (1.21, 2.50), p = 0.003 |
|  |  |  |  |  |  |  |  |
| + multivariable | |  |  |  |  |  |  |
|  | Overall | 1.00 | 1.02 (0.97, 1.08), p = 0.411 | 1.10 (1.01, 1.18), p = 0.023 |  | 1.07 (1.01, 1.13), p = 0.020 | - |
|  | Current abstainer | 1.00 | 1.05 (0.94, 1.17), p = 0.360 | 1.12 (0.97, 1.29), p = 0.136 |  | 1.07 (0.96, 1.18), p = 0.221 | 0.97 (0.85, 1.10), *p* = 0.657 |
|  | <2 g/day | 1.00 | 1.05 (0.96, 1.14), p = 0.286 | 1.12 (0.99, 1.27), p = 0.074 |  | 1.08 (0.99, 1.18), p = 0.077 | Referent |
|  | 2 – 11.99 g/day | 1.00 | 0.95 (0.85, 1.05), p = 0.338 | 1.02 (0.87, 1.19), p = 0.855 |  | 1.02 (0.91, 1.14), p = 0.753 | 0.97 (0.84, 1.11), *p* = 0.663 |
|  | 12 – 23.99 g/day | 1.00 | 1.14 (0.88, 1.48), p = 0.309 | 1.08 (0.73, 1.59), p = 0.709 |  | 1.13 (0.86, 1.46), p = 0.382 | 1.07 (0.82, 1.40), *p* = 0.623 |
|  | ≥24 g/day | 1.00 | 0.90 (0.52, 1.55), p = 0.667 | 1.41 (0.75, 2.66), p = 0.301 |  | 1.23 (0.82, 1.84), p = 0.333 | 1.44 (1.00, 2.09), *p* = 0.051 |

HRs, 95% CIs, and *p*-value derived from Cox models. The multivariable model was adjusted for age, sex, education, marital status, smoking, physical activity, body mass index, resting heart rate, total cholesterol concentration, triglyceride concentration, diabetes, family history of coronary heart disease, history of CVD, and average alcohol intake including current abstainers (if not used as a stratifying variable). Interaction terms were used to test for a slope difference in the relationship between mental health problems (per unit increase of the mental health index) and the outcomes between the groups of alcohol intake, using “<2 g/day” as the reference category. Abbreviations: CVD: cardiovascular disease; HR, hazard ratio; CI, confidence interval.
