# Supplementary material for: Association of coincident self-reported mental health problems and alcohol intake with all-cause and cardiovascular disease mortality: A Norwegian pooled population analysis
Source: PLoS Med. 2020 Feb 3;17(2):e1003030. doi: 10.1371/journal.pmed.1003030 (PMC6996806; doi:10.1371/journal.pmed.1003030)
Supplement: S5 Table — (DOCX) [file pmed.1003030.s009.docx]

|  | | ***Average alcohol intake (grams per day)*** | | | | | | |  |
| --- | --- | --- | --- | --- | --- | --- | --- | --- | --- |
| **HR (95 % CI) in  the sample and  in strata of the  mental health  index** | | **Current**  **abstainer (n = 22,496)** | **Low**  **<2 g/day (n = 85,961)** | **Light**  **2 – 11.99 g/day (n = 116,170)** | **Moderate**  **12 – 23.99 g/day**  **(n =15,944)** | **High**  **≥24 g/day (n = 2801)** |  | **Per 5 g/day  increase among  current drinkers**  **(n = 220,876)** | **Difference in slope (interaction term)** |
| *All-cause mortality* | |  | | |  |  |  |  |  |
| Unadjusted | |  |  |  |  |  |  |  |  |
|  | Overall | 2.01 (1.94, 2.08), p < 0.001 | 1.00 | 0.61 (0.59, 0.63), p < 0.001 | 0.77 (0.72, 0.82), p < 0.001 | 1.40 (1.26, 1.55), p < 0.001 |  | 0.97 (0.95, 0.98), p < 0.001 |  |
|  | 1.00 – 1.50 | 1.92 (1.83, 2.02), p < 0.001 | 1.00 | 0.62 (0.59, 0.64), p < 0.001 | 0.76 (0.70, 0.82), p < 0.001 | 1.20 (1.02, 1.42), p = 0.029 |  | 0.92 (0.90, 0.94), p < 0.001 | Referent |
|  | 1.51 – 2.00 | 2.07 (1.94, 2.22), p < 0.001 | 1.00 | 0.56 (0.53, 0.60), p < 0.001 | 0.73 (0.65, 0.81), p < 0.001 | 1.36 (1.13, 1.63), p = 0.001 |  | 0.95 (0.92, 0.97), p < 0.001 | 1.03 (0.99, 1.06), p = 0.137 |
|  | 2.01 – 4.00 | 2.09 (1.90, 2.30), p < 0.001 | 1.00 | 0.70 (0.64, 0.77), p < 0.001 | 0.86 (0.74, 1.01), p = 0.065 | 1.65 (1.34, 2.03), p < 0.001 |  | 1.06 (1.04, 1.09), p < 0.001 | 1.15 (1.12, 1.19), p < 0.001 |
|  |  |  |  |  |  |  |  |  |  |
| + age and sex | |  |  |  |  |  |  |  |  |
|  | Overall | 1.16 (1.12, 1.20), p < 0.001 | 1.00 | 0.89 (0.86, 0.92), p < 0.001 | 1.05 (0.98, 1.11), p = 0.154 | 1.53 (1.38, 1.71), p < 0.001 |  | 1.04 (1.03, 1.05), p < 0.001 |  |
|  | 1.00 – 1.50 | 1.11 (1.05, 1.16), p < 0.001 | 1.00 | 0.91 (0.87, 0.95), p < 0.001 | 1.01 (0.93, 1.10), p = 0.816 | 1.23 (1.04, 1.45), p = 0.016 |  | 1.01 (0.99, 1.03), p = 0.331 | Referent |
|  | 1.51 – 2.00 | 1.19 (1.11, 1.28), p < 0.001 | 1.00 | 0.84 (0.79, 0.90), p < 0.001 | 1.02 (0.91, 1.14), p = 0.734 | 1.40 (1.16, 1.69), p < 0.001 |  | 1.02 (1.00, 1.04), p = 0.065 | 1.02 (0.99, 1.05), p = 0.180 |
|  | 2.01 – 4.00 | 1.26 (1.14, 1.39), p < 0.001 | 1.00 | 0.95 (0.87, 1.05), p = 0.298 | 1.16 (0.99, 1.37), p = 0.065 | 2.32 (1.87, 2.86), p < 0.001 |  | 1.10 (1.08, 1.12), p < 0.001 | 1.11 (1.08, 1.14), p < 0.001 |
|  |  |  |  |  |  |  |  |  |  |
| + multivariable | |  |  |  |  |  |  |  |  |
|  | Overall | 1.19 (1.15, 1.24), p < 0.001 | 1.00 | 0.93 (0.90, 0.96), p < 0.001 | 1.03 (0.97, 1.09), p = 0.386 | 1.33 (1.19, 1.48), p < 0.001 |  | 1.03 (1.02, 1.04), p < 0.001 | – |
|  | 1.00 – 1.50 | 1.18 (1.12, 1.24), p < 0.001 | 1.00 | 0.95 (0.91, 0.99), p = 0.011 | 1.03 (0.94, 1.12), p = 0.514 | 1.17 (0.99, 1.38), p = 0.069 |  | 1.01 (0.99, 1.03), p = 0.176 | Referent |
|  | 1.51 – 2.00 | 1.24 (1.15, 1.33), p < 0.001 | 1.00 | 0.89 (0.83, 0.94), p < 0.001 | 0.99 (0.88, 1.11), p = 0.841 | 1.25 (1.04, 1.51), p = 0.024 |  | 1.01 (0.99, 1.03), p = 0.375 | 1.01 (0.98, 1.03), *p* = 0.659 |
|  | 2.01 – 4.00 | 1.18 (1.06, 1.30), p = 0.002 | 1.00 | 0.97 (0.88, 1.07), p = 0.511 | 1.09 (0.92, 1.28), p = 0.334 | 1.66 (1.34, 2.06), p < 0.001 |  | 1.06 (1.04, 1.08), p < 0.001 | 1.07 (1.04, 1.10), *p* < 0.001 |
|  | |  |  |  |  |  |  |  |  |
| *CVD mortality* | |  |  |  |  |  |  |  |  |
| Unadjusted | |  |  |  |  |  |  |  |  |
|  | Overall | 2.39 (2.25, 2.55), p < 0.001 | 1.00 | 0.49 (0.47, 0.52), p < 0.001 | 0.57 (0.50, 0.64), p < 0.001 | 0.96 (0.76, 1.20), p = 0.690 |  | 0.85 (0.82, 0.88), p < 0.001 |  |
|  | 1.00 – 1.50 | 2.30 (2.12, 2.49), p < 0.001 | 1.00 | 0.52 (0.48, 0.56), p < 0.001 | 0.56 (0.48, 0.66), p < 0.001 | 0.88 (0.63, 1.24), p = 0.468 |  | 0.81 (0.77, 0.84), p < 0.001 | Referent |
|  | 1.51 – 2.00 | 2.44 (2.18, 2.72), p < 0.001 | 1.00 | 0.45 (0.40, 0.50), p < 0.001 | 0.59 (0.48, 0.73), p < 0.001 | 0.88 (0.59, 1.31), p = 0.517 |  | 0.82 (0.77, 0.87), p < 0.001 | 1.02 (0.95, 1.09), p = 0.643 |
|  | 2.01 – 4.00 | 2.62 (2.22, 3.10), p < 0.001 | 1.00 | 0.53 (0.44, 0.63), p < 0.001 | 0.52 (0.36, 0.75), p < 0.001 | 1.15 (0.73, 1.81), p = 0.543 |  | 1.01 (0.95, 1.06), p = 0.818 | 1.25 (1.16, 1.34), p < 0.001 |
|  |  |  |  |  |  |  |  |  |  |
| + age and sex | |  |  |  |  |  |  |  |  |
|  | Overall | 1.28 (1.21, 1.37), p < 0.001 | 1.00 | 0.82 (0.77, 0.87), p < 0.001 | 0.85 (0.75, 0.96), p = 0.010 | 1.08 (0.86, 1.35), p = 0.513 |  | 0.97 (0.95, 1.00), p = 0.031 |  |
|  | 1.00 – 1.50 | 1.24 (1.14, 1.35), p < 0.001 | 1.00 | 0.85 (0.79, 0.92), p < 0.001 | 0.80 (0.68, 0.94), p = 0.008 | 0.89 (0.64, 1.26), p = 0.534 |  | 0.94 (0.90, 0.97), p < 0.001 | Referent |
|  | 1.51 – 2.00 | 1.29 (1.15, 1.44), p < 0.001 | 1.00 | 0.76 (0.68, 0.85), p < 0.001 | 0.92 (0.74, 1.14), p = 0.454 | 0.92 (0.62, 1.37), p = 0.662 |  | 0.95 (0.91, 1.00), p = 0.032 | 1.01 (0.96, 1.07), p = 0.636 |
|  | 2.01 – 4.00 | 1.40 (1.18, 1.66), p < 0.001 | 1.00 | 0.82 (0.68, 0.99), p = 0.034 | 0.85 (0.58, 1.22), p = 0.376 | 2.04 (1.29, 3.24), p = 0.002 |  | 1.09 (1.05, 1.14), p < 0.001 | 1.17 (1.11, 1.24), p < 0.001 |
|  |  |  |  |  |  |  |  |  |  |
| + multivariable | |  |  |  |  |  |  |  |  |
|  | Overall | 1.25 (1.18, 1.34), p < 0.001 | 1.00 | 0.90 (0.85, 0.96), p = 0.001 | 0.93 (0.82, 1.05), p = 0.257 | 1.04 (0.83, 1.31), p = 0.724 |  | 0.99 (0.97, 1.02), p = 0.606 | – |
|  | 1.00 – 1.50 | 1.25 (1.14, 1.36), p < 0.001 | 1.00 | 0.94 (0.87, 1.01), p = 0.106 | 0.92 (0.77, 1.09), p = 0.310 | 0.95 (0.68, 1.34), p = 0.787 |  | 0.97 (0.94, 1.01), p = 0.125 | Referent |
|  | 1.51 – 2.00 | 1.28 (1.14, 1.44), p < 0.001 | 1.00 | 0.84 (0.75, 0.94), p = 0.003 | 0.96 (0.77, 1.19), p = 0.703 | 0.87 (0.58, 1.30), p = 0.455 |  | 0.96 (0.92, 1.01), p = 0.093 | 0.99 (0.94, 1.05), *p* = 0.821 |
|  | 2.01 – 4.00 | 1.22 (1.02, 1.45), p = 0.029 | 1.00 | 0.85 (0.71, 1.03), p = 0.105 | 0.88 (0.60, 1.27), p = 0.484 | 1.52 (0.95, 2.43), p = 0.078 |  | 1.07 (1.02, 1.11), p = 0.003 | 1.10 (1.04, 1.16), *p* = 0.001 |

HRs, 95% CIs, and *p*-values derived from Cox models. The multivariable model was adjusted for age, sex, education, marital status, smoking, physical activity, body mass index, resting heart rate, total cholesterol concentration, triglyceride concentration, diabetes, family history of coronary heart disease, history of CVD, and mental health problems (if not used as a stratifying variable). Interaction terms were used to test for a slope difference in the relationship between alcohol intake (per 5 g/day increase) and the outcomes between participants with different score on the mental health index. Only current drinkers, and not current abstainers, were included in this analysis. Abbreviations: CVD: cardiovascular disease; HR, hazard ratio; CI, confidence interval.
