# Supplementary material for: Association of coincident self-reported mental health problems and alcohol intake with all-cause and cardiovascular disease mortality: A Norwegian pooled population analysis
Source: PLoS Med. 2020 Feb 3;17(2):e1003030. doi: 10.1371/journal.pmed.1003030 (PMC6996806; doi:10.1371/journal.pmed.1003030)
Supplement: S1 Text — (DOCX) [file pmed.1003030.s010.docx]

This study addresses one of several research questions that constitutes a larger research project. The project has a prospective protocol (in English) describing the overall aim, the specific research questions, data sources, and gives a plan for the analyses (S1 Protocol). In addition, a technical document (only available in Norwegian) gives further details of the data linkage process in particular. The documents were submitted for consideration by an ethical committee and was part of the application for funding to the Norwegian Research Council.

The protocol is not exhaustive in describing all aspects of the study. It was also necessary for several reasons to make some changes before conducting the study, as well as during the peer-review process. Below we describe differences between the planned study as described in the protocol and the conducted study:

***Measure of mental health***: The project aimed to obtain data regarding mental health by linkage to the Social Security Registry (mental, behavioural and neurodevelopmental disorders) and from survey questionnaires providing self-reported mental health problems. Because data linkage to the Social Security Registry was delayed, we decided to conduct the study using self-reported mental health data only.

***Study population:*** The research project was designed and power calculations made on the premise that the source population were surveys within the Cohort of Norway (CONOR) and surveys in the Twin Registry, but the project also includes other data sources. During data cleaning it became clear that some of the surveys that constitute the Age 40 programme could be harmonise with data in CONOR. The inclusion of data form the Age 40 program was a decision made before any analyses in the project, it affects the project as a whole by providing a large boost to the sample size, and obsoletes the power calculations. The Age 40 Program is not described in the protocol, but in the technical document (available in Norwegian). Data on self-reported mental health in the Twin surveys could not be harmonised with data from CONOR or the Age 40 Program. As a result, we did not include data form the Twin surveys in this study.

***Measure of alcohol intake:*** The protocol describes how the drinking frequency was measured in the surveys, and because a measure of drinking frequency was available in most surveys, we planned to use this as the measure of average alcohol intake to get large samples that could power interaction analyses. However, working more closely with the data showed that it was possible to obtain an estimate of the amount of alcohol consumed per day from most of the surveys, and for this particular study, all the surveys had data on the amount of alcohol consumed. As this measure is more accurate than drinking frequency, we solely focus on drinking amount in this study and not the drinking frequency.

The protocol stated to include a subsample with data on binge drinking frequency. Binge drinking was available from some of the surveys in CONOR, from the Twin surveys, but not from the Age 40 Program. However, the need to exclude the Twin surveys resulted in a loss of power, particularly because they were older and provided a larger number of cases per participant in comparison with more recent surveys in CONOR. Because of the loss of power, we decided to drop the focus on binge drinking in the current study, but to focus on the average intake of alcohol.

***Study outcome:*** The protocol planned to study cardiovascular disease mortality, incident acute myocardial infarction and incident stroke. The specific focus on myocardial infarction and stroke were interesting in terms of comparing a binge drinking pattern versus a moderate drinking pattern. By dropping binge drinking from the study (we will addressed the role of binge drinking *per se* in another paper), we decided to change the outcome. We decided before data analysis to study cardiovascular disease mortality and all-cause mortality. The reason for including all-cause mortality is that this outcome could encapsulate all the potential interactions between alcohol and mental health.

***Statistical analyses*:** The analysis plan for time to event outcomes was to use Cox proportion hazard regression models stratified by mental health, either operationalised as mental distress or depressed/not depressed, and to test for interaction (although this was stated more clearly for other related research questions). We followed this plan, and also elaborated on the interaction analyses based on the suggestions in Knol MJ & VanderWeele TJ (2012) Recommendations for presenting analyses of effect modification and interaction. Int. J. Epidemiol. 41(2):514-520).

We initially performed the analyses using a dichotomised cut-off for distinguishing those who were distressed (above a mean score >2.15) versus those who were not distressed (<2.15). However, we later decided to change the operationalisation to a more graded assessment using three groups. This decision was not based on results of the first analyses. Mental health is not black and white, and since we were using a graded measurement and not clinical diagnoses, we wanted our analyses to reflect this.

***Research question:*** The protocol stated to: *“Investigate if the effects of moderate alcohol consumption and binge drinking on CVD can be explained by or interact with mental distress (depression and/or anxiety)”*. Because of the changes made, we rephrased the aim of the study. In the abstract, we now state that “*The objective of this study was to investigate cardiovascular disease (CVD) and all-cause mortality according to mental health problems and alcohol intake in the general population*”. In the manuscript text, we elaborate on this objective by being more specific in order to help the reader what type of analyses we made.
