# Supplementary material for: Association of coincident self-reported mental health problems and alcohol intake with all-cause and cardiovascular disease mortality: A Norwegian pooled population analysis
Source: PLoS Med. 2020 Feb 3;17(2):e1003030. doi: 10.1371/journal.pmed.1003030 (PMC6996806; doi:10.1371/journal.pmed.1003030)
Supplement: S3 Text — (DOCX) [file pmed.1003030.s012.docx]

Questions used to create the mental health index (score):

| Did you in the past two weeks feel? | | | | | |
| --- | --- | --- | --- | --- | --- |
|  | No (1) | A little (2) | Quite a bit (3) | Very (4) |  |
| Nervous and unsettled |  |  |  |  |  |
| Troubled by anxiety |  |  |  |  |  |
| Secure and calm |  |  |  |  |  |
| Irritable |  |  |  |  |  |
| Happy and optimistic |  |  |  |  |  |
| Sad/depressed |  |  |  |  |  |
| Lonely |  |  |  |  |  |

Example 1:

| Did you in the past two weeks feel? | | | | | | |
| --- | --- | --- | --- | --- | --- | --- |
|  | No (1) | A little (2) | Quite a bit (3) | Very (4) | Score |  |
| Nervous and unsettled |  |  |  | X | 4 |  |
| Troubled by anxiety |  | X |  |  | 2 |  |
| Secure and calm |  |  | X |  | 2* |  |
| Irritable |  |  |  | X | 4 |  |
| Happy and optimistic |  |  | X |  | 2* |  |
| Sad/depressed | X |  |  |  | 1 |  |
| Lonely | X |  |  |  | 1 |  |
| Sum (range 7 – 28) |  |  |  |  | 16 |  |
| Mean (range 1 – 4) |  |  |  |  | 2.29 |  |

* Score order reversed for “secure and calm” and “happy and optimistic”.

Example 2:

| Did you in the past two weeks feel? | | | | | | |
| --- | --- | --- | --- | --- | --- | --- |
|  | No (1) | A little (2) | Quite a bit (3) | Very (4) | Score |  |
| Nervous and unsettled |  |  |  | X | 4 |  |
| Troubled by anxiety |  | X |  |  | 2 |  |
| Secure and calm |  |  | X |  | 2* |  |
| Irritable |  |  |  | X | 4 |  |
| Happy and optimistic |  |  | X |  | 2* |  |
| Sad/depressed | X |  |  |  | 1 |  |
| Lonely | Na | Na | Na | Na | §1.18 |  |
| Sum (range 7 – 28) |  |  |  |  | 16.18 |  |
| Mean (range 1 – 4) |  |  |  |  | 2.31 |  |

* Score order reversed for “secure and calm” and “happy and optimistic”.
§ Mean value in the study population to replace 1 missing value.
