# Supplementary material for: Association of coincident self-reported mental health problems and alcohol intake with all-cause and cardiovascular disease mortality: A Norwegian pooled population analysis
Source: PLoS Med. 2020 Feb 3;17(2):e1003030. doi: 10.1371/journal.pmed.1003030 (PMC6996806; doi:10.1371/journal.pmed.1003030)
Supplement: Attachment — Submitted filename: degerund.pdf [file pmed.1003030.s014.pdf]

Review of PMEDICINE-D-19-02500R1

October 26, 2019

Overall, this revision was very responsive to my previous comments. A few issues remain and are outlined below.

1 Major Comments

1. Thank you for providing detailed information about the collection of surveys that gave rise to the study data. However, I still do not understand how the one visit per individual was chosen (as mentioned on line 130) if individuals had more than one "visit" in any of the data sources? This is a concern because differences in the timing of selected visits by exposure group could confound the results (which is one reason to control for age, as the authors point out). This should be clarified.

2. How were covariates included in the regression models? For example, was age modeled as a linear variable, using categories, or another type of flexible functional form? How were functional forms chosen?

2 Minor points

1. Line 239: the paper notes that HDL-C and blood pressure were considered mediators of the alcohol-mortality relationship. The following sentence seems to indicate that these variables were considered confounders of the mental health-mortality associations, and therefore included in the models for mental health. How were these variables handled in the models for the joint effects?

2. Line 240: the paper states that the multivariable model treats "these variables" as confounders while the model adjusted for age and sex

treats them as mediators. This section is unclear. Do the authors consider these variables to be mediators or confounders? This section should be rewritten to reflect that the scientific question and knowledge of the causal structure determines the models used, and not vice versa.

3. Throughout, the paper refers to “beta” coefficients. While this is common language in biostatistics, it may be preferable to refer to regression coefficients to prevent confusion.

4. In the methods or results, the paper should indicate the maximum follow-up time. In other words, over what time period are the reported hazard ratios applicable? As noted in Hernn MA. The hazards of hazard ratios. Epidemiology (Cambridge, Mass.). 2010 Jan;21(1):13, the hazard ratio is sensitive to the time period chosen.

5. Line 467: “higher” than what?

6. In the “methodological considerations” section of the Discussion, the authors may also want to discuss the fact that, to be included in the study, participants had to survive until mid-life, when the surveys were implemented. Accordingly, results may not hold for the effects of drinking and mental health at earlier ages.
