# Supplementary material for: Association of coincident self-reported mental health problems and alcohol intake with all-cause and cardiovascular disease mortality: A Norwegian pooled population analysis
Source: PLoS Med. 2020 Feb 3;17(2):e1003030. doi: 10.1371/journal.pmed.1003030 (PMC6996806; doi:10.1371/journal.pmed.1003030)
Supplement: Attachment — Submitted filename: Response to reviewers.docx [file pmed.1003030.s013.docx]

# Rebuttal letter

Dear external reviewers and editors at PLOS Medicine.

On behalf of the authors, I thank you for conducting a thorough review and critical appraisal of our manuscript. We really appreciate the time that you have invested. The questions and comments were clear, constructive and very helpful.

Among the points raised, we were asked to shed more light on what might be going on to cause an interaction between mental health problems and alcohol intake on mortality, and to indicate the use of psychotropic drug use in the study population. Furthermore, a more detailed description of the source population and sample size selection was warranted, and the reporting of the methods and results needed improvement. We were also asked to provide an author summary and an additional paragraph in the discussion. In addition, we have made minor changes to the manuscript that can be identified from the manuscript with track changes enabled.

We hope that the reviewers and editors find the revised version of the manuscript improved. If you disagree with our arguments or decisions, have further questions, comments, or concerns, we would appreciate the opportunity to address them and make further revisions if needed. The revised version is longer than the previous manuscript as a result of the peer-review. We have tried to be clear and concise, but we acknowledge that there will always be ways to condense a manuscript even more if this is required. In the remainder of this rebuttal letter, we address each specific point made by editors and reviewer.

October 16, 2019.

Corresponding author

Eirik Degerud

# **Editors:**

1. **Thank you for providing a prospective protocol (S1_protocol). Please note the date the protocol was written, and note in the text any instances where analyses differed from the protocol, and reasons for any such changes.**

   We have added the date the protocol was written at the top of the first page in the protocol (S1_Protocol). We have in a supplemental file (S1 Text) provided an overview of differences between the study performed and the study planned as described in the protocol.

   In the main text, we refer to this file in the Methods:

   #147 “The study is part of a larger research with a prospective protocol (S1 Protocol). We provide an overview of differences between the conducted study and the planned study as described in the protocol (S2 Text).
2. **Thank you for the note regarding your data-sharing restrictions. However, PLOS Medicine requires that the de-identified data underlying the specific results in a published article be made available, without restrictions on access, in a public repository or as Supporting Information at the time of article publication, provided it is legal and ethical to do so. Please see the policy at:**
   [**http://journals.plos.org/plosmedicine/s/data-availability**](http://journals.plos.org/plosmedicine/s/data-availability) **and FAQs at:** [**http://journals.plos.org/plosmedicine/s/data-availability#loc-faqs-for-data-policy**](http://journals.plos.org/plosmedicine/s/data-availability#loc-faqs-for-data-policy) **In addition, please note that an author cannot serve as primary contact for obtaining the data.**

   We thank the editors for the comment as well as the link to the PLOS Policy on how to report on data sharing. The note regarding data-sharing has been updated and we hope that it has improved. We chose a short and clear note without too many details, but if PLOS policy or the editors require a more elaborate note that gives more details regarding the ethical or legal issues, or more of the details that we left out from previous version, we will provide this upon request immediately.

   *“The results in this study are based on de-identified data from human research participants. Data can be made available to all interested researchers upon request. For more information, please contact the Norwegian National Institute of Public health, Division of Health data and Digitalisation (lhu@fhi.no). The reason why the data are not made publicly available in a public repository or as a supporting information is because of local legal restrictions as well as ethical restrictions related to privacy. The participants have not consented to their data becoming publicly available and they might lose control of their data, such as their right to have their data deleted.”*
3. **Abstract: Please include number of participants included in the study.**

   The abstract now states the number of participants included in the study (#31).
4. **Abstract and throughout: Please include p-values along with 95% CIs.**

   We have included p-values along with 95% CIs in the abstract and throughout.
5. **Abstract: At Line 51 There is a grammatical issue: “less” should be “fewer”.**

   We removed this sentence in the abstract, but made a correction in the main text.
6. **Abstract: Please move the limitation sentence (last sentence of abstract’s Conclusion section) to be the last sentence of the abstract’s Methods and Findings section.**

   We have moved the sentence to be the last sentence in the abstract’s Methods and Findings (#51).
7. **Author Summary: At this stage, we ask that you include a short, non-technical Author Summary of your research to make findings accessible to a wide audience that includes both scientists and non-scientists. The Author Summary should immediately follow the Abstract in your revised manuscript. This text is subject to editorial change and should be distinct from the scientific abstract. Please see our author guidelines for more information:** [**https://journals.plos.org/plosmedicine/s/revising-your-manuscript#loc-author-summary**](https://journals.plos.org/plosmedicine/s/revising-your-manuscript#loc-author-summary)

   An Author summary has been included in the revised manuscript immediately following the Abstract. Our goal is to make the study as available as possible to the reader. We welcome any editorial changes that improve the communication of the study and also acknowledge that the interaction is open for interpretation in terms of the mechanisms involved.
8. **Methods and Results: A table describing cohort demographics (such as table S1) should be found in the main text.**

   We have interpreted this comment as a request to include in the main text a table providing descriptive characteristics of the study population according to categories of mental health problems and alcohol intake. *A traditional table 1*. In the previous version of the manuscript, this information was provided in the supplemental data (as Tables S2 and S3). Accordingly, we have assumed that the reference to “Table S1” was a typo. In the revised version, we have made a selection of the most important and informative variables, and present them in Table 1. Additional variables are presented in Table S3. We think two tables are better, simply because the amount of information would be too much for a single table. However, we are more than willing to reconsider changing the lay-out or the selection of variables if the editor disagrees with the current lay-out. We also add that many of the tables have a smaller font size than in the main text. If there are any issues related to this, we will try to find alternative ways of formatting or arranging the table. A request to include in the main text “*a traditional Table 1*” was also made by reviewer number 3.

   Lastly, there is a possibility that we have misinterpreted the editors. The editors might have been asking to include in the main text a table providing demographic data of the different surveys constituting the source population. This would mean that the reference to Table S1 was not a typo. If this is the case, then we apologize, and hope we get the chance to make the appropriate revisions.
9. **Methods and Results: Please add the following statement, or similar, to the Methods: "This study is reported as per the Strengthening the Reporting of Observational Studies in Epidemiology (STROBE) guideline (S1 Checklist)."**

   We have added the following statement at #144:

   “This study is reported as per the Routinely Collected Health Data (RECORD) guideline, which is an extension of the Strengthening the Reporting of Observational Studies in Epidemiology (STROBE) guideline (S1 Checklist).”

   Please see our reply to comment number 23 regarding the choice of guideline.
10. **Methods and Results: Survey response rates were not included. Please include survey response rates.**

    In the revised manuscript, we have included survey response rate in **Table S1**. The response rate varied from 37.5% to 78%. In the main text, we state this response rate range in the method section (#125-129) and discuss what it could mean in terms of generalizability in the Discussion in the paragraph “Methodological considerations” #512-516.
11. **Methods and Results: Line 96-98: There appears to be a typo “The Regional Ethics Committee South-East (11/1676) approved the study and gave exemption regarding consent in surveys were this was not obtained.” (were should be where)**

    Corrected in the revised manuscript.
12. **Methods and Results: Please provide p-values for HRs and CIs reported in Table 1 and Table 2.**

    The revised manuscript provides p-values for HRs and Cis reported in Tables 2, 3, S4, and S5.
13. **Methods and Results: Results from models adjusted for age and sex are presented, as well as multivariable models. Please also present results from unadjusted models.**

    The revised manuscript now contain the results from unadjusted models without age and sex in the main text and in supplemental data (Tables 2, 3, S4 and S5). To accommodate unadjusted models and p-values (as requested in comment #12), we decided to reduce the font size to condense the information into the standard word-format. We hope that this is appropriate, but if not, we will find another way of tabulating the information.
14. **Methods and Results (and supplemental data): Line 332: “Gender” is used, while “sex” is used elsewhere. Both terms are used in the supplemental information. The terms gender and sex are not interchangeable (as discussed in** [**http://www.who.int/gender/whatisgender/en/**](http://www.who.int/gender/whatisgender/en/) **); please use the appropriate term.**

    Very true. In the revised manuscript, we correctly only use sex throughout the manuscript and supplemental information.
15. **Discussion: Between the limitations section and conclusions section, please add a brief discussion of implications and next steps for research, clinical practice, and/or public policy.**

    The revised manuscript now includes a discussion of the implications of the study findings as well as a discussion of the next steps for research in light of the limitations of the current study (#522 – 543). We hope that the content is relevant to the request and that it’s not too long. We would like to add that we have have moved information from other parts of the discussion to this new paragraph.
16. **Figures 1, 2, and 3: It is difficult to see the difference between the blue and black dashed lines.  Please make this difference more clear (e.g. change the colors, line weights, etc.).**

    We have changed the colors in the revised manuscript to make the difference more clear.

    Figures 2 and 3 have been renamed as Figures 2A and 2B because they are identical a part from showing the functional form of the association with different outcomes.

    Note to the editors. Reviewer #3 has raised some concern about the added value of showing these figures. This is a valid point. The formal assessment of the associations are based on the comparison of hazard ratios in the tables (Table S4 and S5 in the revised manuscript). Our argument for showing them, which we present to reviewer 3, is mainly pedagogical. By looking at the figures, the reader get a quick feeling for the relationships (such as whether they are linear or non-linear, any difference between the curves, the degree of overlap between the CIs, and where there are more or less data points). This is also how we use them in the revised version of the manuscript. We are willing to reconsider the inclusion or placement of the figures if the reviewer or editor considers them to be redundant.
17. **Figure S3: For the Kaplan-Meier curve, please provide the number at risk for each time interval.**

    We decided to remove Kaplan-Meier curves from the manuscript. The main rational for including the curves in the previous version was to provide a crude visualization of the associations, to inspect for any violation of the assumption of proportional hazards, and to add a crude test of whether the survival probability differed according to alcohol intake and mental health using the log-rank test. They were never the primary analysis for testing the study objectives. Now that we have included unadjusted hazard ratios in Tables S4 and S5, which provides a crude assessment of the separate associations, we think that the curves are less important. Reviewer 3 has also encouraged us to consider whether all of the different analyses are justified. If the editor disagrees with this decision, we are willing to reconsider. We did perform the suggested changes to the Kaplan-Meier curves (comment 17 – 20) and have the revised figures available.
18. **Figure S2, and S3: Please show the axis beginning at zero. If this is not possible, please show a break in the axis.**


    For figure S2 (relationship between alcohol intake and the mental health index), starting the axis at the value 1 (corresponds to zero because the range is 1 to 4) if feasible and is usually not a problem to do technically. Unfortunately, the only way we are able to expand the lower end of the vertical axis (down to 1) is if the higher end is also expanded (up to 4). This does not look good. When setting the vertical axis upper limit to the value 2.3, the software (R + ggplot2) kicks out the data between 2.3 – 4, which is also not a good solution. Consequently, we have added a break in the axis and write in the figure legend that the vertical axis is truncated. We did try alternative plotting commands, but this setup was the only one we found that allowed us to include both current abstainers as a separate group and the smoothed mean for drinkers.

    For figure S3: We decided to remove the Kaplan-Meier curves. Please see our reply to comment number 17.
19. **For Figure S3: Please indicate in the figure caption the meaning of the dotted lines vs. solid lines. In the legend of figure S3, p-value = 0 should be p-value < 0.001.**

    We decided to remove the Kaplan-Meier curves. Please see our reply to comment number 17.
20. **For Figure S3: Panel B legend has “Alcohol intake” at the top- remove for consistency, or add a description of different plotted series to graphs in panels A, C, and D.**

    We decided to remove the Kaplan-Meier curves. Please see our reply to comment number 17.
21. **Table S4: The legend has a typo- a period is missing at the end of “HR and 95% CI and p-value derived from Cox models”.**

    Corrected.
22. **References: Please use the "Vancouver" style for reference formatting, and see our website for other reference guidelines:** [**https://journals.plos.org/plosmedicine/s/submission-guidelines#loc-references**](https://journals.plos.org/plosmedicine/s/submission-guidelines#loc-references)

    The revised manuscript applies the “PLoS” output style from Endnote for reference formatting.
23. **Thank you for submitting the STROBE checklist.  Please confirm that STROBE is the most appropriate reporting guideline, rather than RECORD.  Please report your study according to the relevant guideline, which can be found here:** [**http://www.equator-network.org/**](http://www.equator-network.org/)

The study did not fit entirely with the typical study targeted by the RECORD checklist. However, some of the information requested in RECORD and not in STROBE are indeed relevant for the reporting of this study. Another argument is that RECORD works as an extension of STROBE, not a different alternative. Accordingly, we have submit the RECORD checklist together with the revised manuscript. In order to report the study according to the RECORD checklist, we needed to include in the revised manuscript some more information on data linkage.

# **Reviewer #1:**

**This paper reports findings from an analysis of combined national survey data linked to mortality in which the authors investigated the association between self-reported level of alcohol intake and mortality and the extent to which this varied according to concurrent reported mental health status. The methodological approach is robust, in my opinion, the paper is well-written, and the authors draw appropriate conclusions. My comments are as follows:**

1. **Although unlikely in an unselected community sample, I suppose it is possible that the modifying effect is of psychotropic medication rather than mental health status. It would have been helpful to have had some indication of the level of psychotropic medication use in people within the different mental health status groups, even if this couldn't be considered as a covariate. If this is not possible then I feel it needs to be highlighted as a potential limitation.**

   We agree with the reviewer that this would be relevant information. We have gone through the questionnaires applied in the source surveys to look for self-reported information. Information regarding use of antidepressants and tranquilizers in the last 4 weeks (Not used, daily, weekly but not daily, and less than weekly) was available in four surveys in CONOR (HUBRO, OPPHED, TROMSØ V, TROFINN), but not all the surveys in CONOR, and not at all in the Age 40 Program. Another question asked whether the person had ever sought help for psychological problems, and this was available from all surveys in CONOR, but not the Age 40 Program.

   The number of participants with information on psychotropic drug use (n = 27.000) is, in our opinion, too low to power a sensitivity analysis of any kind. In the revised manuscript, we have therefore followed the reviewer’s suggestion to indicate the use of these medications in the different mental health status groups by providing this information for the subsets. We mention psychotropic drugs in the introduction (#104), describe the measurements in the method section (#198-200), present the values in “Table 1” and summarise them in the result section (paragraph “Participant characteristics” #295-298 and #309-313) and discuss them in the paragraph “Methodological considerations” #493-497, and finally in a new paragraph “Implications and future research” “#533-537 as one of several potential explanation for our findings .

   Quoting:
   *#*493-497*“..and in a subset we found a higher use of antidepressant and tranquilizers among current abstainers, as well as people reporting high intakes of alcohol and people with higher scores on the mental health index.”*

   #533-537 *“Unfortunately, the study design does not shed light on the exact mechanism underlying the interaction. A longitudinal study design could provide more answers, especially if it includes repeated measurements of mental health problems, alcohol intake, and health data related to risk factors, health behaviour, dietary factors, and the use of psychotropic drugs.”*
2. **I found the text a little confusing on what was done about people who had participated in more than one survey. On p10 it is implied that they were included, but it is not clear (beyond data availability) how one survey point was chosen over another. And then on p15 it is implied that participants in multiple surveys were excluded, which seems to contradict the earlier statement. Some clarification of text is needed.**

   The selection process should be understandable if communicated clearly. We agree that this was not the case in the submitted paper and have tried to improve on this in the revised manuscript. This includes the paragraph in the method section “Study population, data linkage, ethical approval, and selection” and the paragraph in the result section “Study population”.

   In brief, the Cohort of Norway and the Age 40 Program are both large cohorts made up of different health surveys. In the Cohort of Norway, two surveys from the Tromsø Study were included (wave number 4 and 5). Because many people in Tromsø 4 were re-invited to participate in Tromsø 5, there is a considerable degree of overlap (n = 7017). In the revised version, we clearly state this and that we chose to include the first visit (Tromsø 4).

   In addition, there is some overlap between the Cohort of Norway and The Age 40 Program (albeit not within the Age 40 Program). The revised version of the manuscript now clearly states that we placed priority on observations from the Cohort of Norway (this was arbitrary), but if a relevant individual had missing values on alcohol status in the Cohort of Norway and data on drinking status from the Age 40 program (n = 134), we included the observation from the Age 40 Program.
3. **On P16, lines 231-234, it would be helpful if the authors coud clarify for readers what's meant by 'index score' (I assume it is the mental health measure - if so, could it be referred to as this?).**

   Thank you for pointing this out. You were correct in assuming that we meant the mental health measure. Or “the mean score per question on the mental health index”, to be precise. We have made this clear in the revised manuscript. There are also other places in the manuscript where we improve on the reporting. However, we do write only “index score” in some cases when the word is repeated very frequently within a paragraph
   .
4. **The labelling of different models for rows in Table 1 (p23, lines 315-316) seems to be missing. The same issue applies to Table 2.**

   We apologize for this error that probably occurred when embedding the tables in the manuscript text. The labels were there, but hidden because of a lack of row length. We have fixed this in the revised manuscript.

# **Reviewer #2:**

**This manuscript describes associations of measures of mental health and alcohol consumption with both all-cause mortality and CVD mortality using pooled data from Norwegian health surveys conducted between 1994 and 2002. Linkage to death registration covered mortality up to the end of 2014. The total source population for the study was over 300,000 individuals and over 21,000 had died by 2014 (6,587 from CVD). Cox Proportional Hazards Models were used for the main analyses to describe the independent associations of mental health and alcohol consumption with mortality, and the interaction terms between mental health and alcohol consumption, adjusted first for age and sex only, and then for age, sex, education, marital status, smoking, physical activity, body mass index, resting heart rate, and total cholesterol. The pattern of findings indicated higher mortality at higher bands of mental health index score (all-cause and CVD but less so for the latter); higher mortality for other groups than for the low average alcohol daily intake group (<2 g/day) with increasing mortality at higher levels of current drinking; and an interaction whereby increasing alcohol intake was more strongly associated with increased mortality in groups scoring more highly on the mental health index. This was most evident for the subgroup with high mental health score and high alcohol intake (>=24 g/day) that had very high mortality rates. The patterns were similar when additional covariates were included in the models but the magnitude of differences was attenuated. The pattern of interaction was also shown by expressing the HR per 5g/day increase in consumption within strata of mental health index score, where the HR for the highest stratum was greater than for the two lower mental health strata.**

**The paper has strengths, especially in terms of the available data and size of population. This facilitates the quantification of the identified interaction. The manuscript also acknowledges limitations, especially the cross-sectional nature of the risk factor data. However, it also presents and discusses the findings in terms of a model of "susceptibility" to the health risks of drinking, where those with more mental health problems have increased susceptibility. This interpretation is the greatest weakness of the manuscript and it evolves from a narrative that is very sparse in terms of providing an account of what mechanisms might underlie the key observations. There are glimpses of what the authors might be thinking, such as the mention in the abstract that mental health problems "might become worse from drinking" but this does not constitute a coherent explanation or hypothesis for future investigation. There is also a statement at lines 172-173 that HDL-C and systolic blood pressure "are likely to play a mediating rather than a confounding role" but it isn't explicit what association/pattern they are mediating. Further, there is a mention of "genetic risk for alcohol addiction" (line 395) in the Discussion. There could well be other plausible accounts of the reported interactions and one possible explanation is that groups with high mental health scores and high current drinking have a longer history of heavy use. Overall, the paper presents an intriguing and potentially useful empirical finding but sheds too little light on what might be going on to bring it about.**

We are glad that the reviewer appreciates the strength in the data and finds the empirical findings intriguing and potentially useful. We are also thankful for the constructive criticism and suggestions on how to improve the manuscript. The reviewer calls for us to shed more light on the mechanisms that might be going on to bring this interaction about. We agree that this was sparsely covered and somewhat scattered in the previous version, and hope that the reviewer finds that the revised version of the manuscript has improved in this regard. However, we acknowledge that there could be several candidate mechanisms not mentioned in the revised manuscript, or that some mechanisms could be more important. Given the vast literature and extensive research on the topics of alcohol and mental health, we appreciate any additional comments or suggestions on how to interpret this interaction from the view of the experienced reviewer.

In the introduction of the revised version, we describe and elaborate more on the different mechanism linking alcohol intake and mental health problems (the bi-directional relationship and shared environmental and genetic factors). We highlight that these mechanisms could lead to a more severe and extreme phenotype and that this could increase the mortality risk over time. We also mention other mechanisms that could be involved, such as the susceptibility hypothesis, other risk factor such as psychotropic drugs (another reviewer’s suggestion), and that changes in the environment over time could modify the mortality risk.

Please see #92 – 100 for mechanisms explaining the relationship between alcohol and mental health and #101 – 108 for how these mechanism might be linked to an increased mortality when they occur together.

In the discussion, we have included a new paragraph labelled “Implications and future research” on the editors request where we pick up the thread from the introduction. The cross-sectional nature of the study design prevents us, as the reviewer points out, from revealing the exact mechanism involved. We use this as a basis for suggesting what type of data or studies that might provide more answers in the future.

Quoting:

#532 – 543: *Unfortunately, the study design does not enable us to clarify the exact mechanism underlying the interaction. A longitudinal study design could provide more answers, especially if it includes repeated measurements over the life course of mental health problems, alcohol intake, and health data related to risk factors, health behaviour, dietary factors, and the use of psychotropic drugs. Preferably, also data on drinking patterns. Such as study could compare the risk associated with different sequence of events (a high alcohol intake preceding mental health problems or vice versa) and determine whether specific combinations is associated with a progression into more extreme and adverse phenotypes. Genotyping of large cohorts harbouring relevant information, such as the Nord-Trøndelag Health Study (The HUNT Study), could reveal the role of genetic variants predisposing individuals to both alcohol use and mental health problems over time.”*

Replies to specific comments included in the main comment:

**Quoting: *“There is also a statement at lines 172-173 that HDL-C and systolic blood pressure "are likely to play a mediating rather than a confounding role" but it isn't explicit what association/pattern they are mediating****.”*

In the revised manuscript, we explicitly specific that they are considered to be mediators of an effect of alcohol intake on CVD mortality. See lines #236-242.

**In addition to this main point there a number of more specific points for the authors to consider.**

1. **The mention in the introduction (lines 63-64) of a positive association between alcohol intake and mental health gets into an unnecessary debate. There are a number of studies (not cited) that find poorer mental health in abstainers compared with light drinkers. However, this has very little to do with the ingestion of ethanol* and such J or U shapes (including this study's findings in Table S2) are not a threat to the core analyses of the present research. I note, too, that reference 7 appears to be cited to support both sides of the above debate. (I haven't checked to see whether it does have ambiguous results.)**

   We thank the reviewer for providing the study in question, and not only stating that there is literature that we should have referred too. We wrote in the previous manuscript: “*There is a positive association between alcohol intake and mental health problems in most [6-8], albeit not all observational studies [7, 9]*”. If we interpret the reviewer correctly, the “unnecessary debate” is that we are debating whether there is a non-linear relationship, when it is already well-known that abstainers deviate from current drinkers. In the revised manuscript, we have revised the sentence above in light of our understanding of the comment and after reading the suggested reference (* Lucas, N., Windsor, T. D., Caldwell, T. M. & Rodgers, B. (2010). Psychological distress in non-drinkers: Associations with previous heavy drinking and current social relationships. Alcohol and Alcoholism 45 (1), 95-102.). In addition, based on another reviewer’s request, we present in the revised manuscript the distribution of the use of tranquillizers and antidepressants in a subset of the study population. We find that current abstainers and people with high alcohol intake (>24 g/day) report the highest use of these drugs, supporting the U or J-shaped distribution of the mental health index score over drinking categories. In the discussion, we link the findings of higher use of psychotropic drug use among abstainers with the suggested reference above.

   Places in the manuscript were the additional data on psychotropic drug use is mentioned: Introduction (#104), methods (#198-200), results (“Table 1” and paragraph “Participant characteristics” #295-298 and #309-313), discussion (paragraph “Methodological considerations” #493-497 and paragraph “Implications and future research” “#533-537).

   We also thank the reviewer for identifying the error related to reference 7 being cited twice.
2. **I think more information is needed in the Methods (lines 87-88) about the surveys other than the Age 40 Program rather than readers having to look elsewhere (ref. 16). What age ranges are included? I would be uncomfortable if these other data sets included younger adults where the nature of non-CVD deaths could be very different from those found at older ages. I'm looking for some reassurance here.**

   We have restructured the method section in the revised manuscript. Please see the paragraph “Study population, data linkage, ethical approval, and selection”. In brief, the section now starts with an introduction to the Age 40 Program and the Cohort of Norway. We then summarize the surveys identified from the Age 40 Program and Cohort of Norway, such as the year the survey was performed, the age range and sex distribution. The supplemental table providing demographic statistics on each health surveys (Table S1) now also include response rates for each survey.

   The reviewer also points out that the nature of non-CVD deaths differs between people in high and low ages, and is concerned about how this may relate to the study. Below is a histogram depicting the distribution of “age when attending the health survey”. There is a dominance of people aged between 35 and 50 (73% to be precise), but the study population does include both younger and older adults.

   Although the most important part was to identify that there is an interaction occurring in the general population, we agree that the inclusion of a wide age group makes it difficult to narrow down the search for potential mechanisms. The interaction could for example be more or only relevant for a particular age group, and indeed, the nature of the CVD deaths could differ.

   We have performed a sensitivity analysis were we repeated the analyses that formed the basis of Tables 3 and 4 (joint association) in a subset of people aged between 35 and 50 years of age when attending the survey. In practice, we include individuals included from the Age 40 Program as well as some of the participants in CONOR. For all-cause mortality, the results in the subset are similar to the findings in the full sample, which was the primary concern of the reviewer. We hope that this is reassuring. The only difference was that the point estimates (HRs) tend to become slightly more pronounced in some categories. For CVD mortality, however, we lose statistical power by excluding those above 50 years of age, and it is not possible to draw any conclusions from the sensitivity analysis.

   We have not included this sensitivity analysis in the revised manuscript, but if the reviewer insists, we are willing to reconsider. We have however stated the age range more clearly in the method section, as well as mention the age range as a factor that might influence the external validity of the study in the discussion under “Methodological considerations”. Please see line numbers #503 – 520. We thank the reviewer for this important comment.
3. **There were times in the descriptions of the analyses where a particular analysis appeared to be limited to current drinkers but I couldn't always follow why this was so and under what circumstances it was necessary. Current abstainers are included in the tables, and the figures showing the Kaplan-Meier risk curves (Figs. 2 and 3) have lines starting at 0g/day. Can it be made clearer which analyses excluded current abstainers and why?**

   This should indeed be crystal clear and we have tried to improve on this in the revised version of the manuscript. Current abstainers are excluded in analyses were we model the average alcohol intake (grams/day) as a continuous variable in Table S5 and in the plots depicting the functional form of the associations with the log hazard ratio on the y-axis (Fig 2A and 2B).
4. **The early parts of the Discussion are quite vague in expressing the quantification of effects. "Increased risk", "more susceptible", "higher" and "considerable" are descriptions which give little idea of whether the differences found are large or small. The Discussion also suffers particularly from the main weakness of the paper as outlined above.**

   We have revised the paragraph “Brief summary” to express the quantification of effects. As an example, we now write that *“In joint analyses, we observed a 129 % and 78% higher risk of all-cause and CVD mortality for the combination of a high alcohol intake and high mean score on the mental health index (compared with a low index score and a low alcohol intake). These estimates were 64 – 69 % higher than expected under the assumption of a multiplicative interaction structure.” With regards to the remainder of the discussion, please see our reply to the main comment.*
5. **The number of participants for the HUBRO Cohort is reported incorrectly in Table S1.**

   Thank you. We have corrected this error in the revised manuscript.

# **Reviewer #3:**

**This paper addresses the important topic of interaction between alcohol use and mental health status on all-cause and CVD-related mortality. However, the paper has several issues that limit the interpretability of the findings:**

1. **Presentation of methods and results: The statistical methods and results sections were poorly organized and difficult to follow. The authors present stratified smoothed hazard functions (in the figures), hazard ratios for the joint effects of alcohol use and mental health status, Kaplan Meier plots, and log rank tests. It is unclear in the methods and results sections why all of these different types of results are presented (e.g., which hypothesis is addressed by each set of results).**

   We have re-organized parts of the method and result section to try to improve on the reporting. Especially we made revisions in the paragraph “Statistical analyses” in the method section to more clearly relate the study objectives to the specific HRs assessed and to explain the purpose of performing different analyses. In the result section, we describe more clearly what the different results provide.

   We also state the rational for including the smoothed log hazard ratio plots, which was primarily for visualization purposes to help the reader. The Kaplan-Meier plots and the log rank test was included as a visualization as well as a crude test of the first objective (separate associations). However, having discussed their relevance in different type of observational studies (RCTs versus cohort studies), and having included unadjusted HRs (editors request) in tables 2, 3, S4 and S5, we have decided to remove the Kaplan-Meier plots and the log rank test.

   More details are included in our replies to the specific comments made by the reviewer below. We hope the reviewer finds the manuscript improved. If the reviewer disagrees with our decisions or have new questions, we hope to get the chance to make additional improvements. We thank the reviewer for taking the time to read our manuscript thoroughly and for providing very clear and constructive comments on how to improve the presentation of methods and results.
2. **Interaction vs effect modification: In the main text, the paper presents joint hazard ratios, which indicate that the authors are interested in interaction between alcohol use and mental health. However, the primary conclusion seems to be that alcohol is worse for people with worse mental health, which seems to indicate that they are interested in describing the different effects of alcohol within strata of mental health status. (in the former case, joint hazard ratios (with a common referent group, are logical to estimate joint effects; in the latter case, one would present hazard ratios for alcohol use within strata of mental health status). The wording of the conclusion should be consistent with the methods and results.**

   The reviewer is correct. The conclusion in the abstract and the main text should be consistent with the primary focus on the joint HRs and interaction, and not a one-sided focus on alcohol with mental health as an effect modifier. We have made changes in the revised version of the manuscript to fix this. However, there are many potential mechanism that may be underlying the statistical interaction, some of which are more in line with interaction, and some that would be more in line with effect modification.

   We have also made revision to make it clear why we estimate separate, stratified and joint HRs. We highlight that this set-up for interaction analyses was informed by a paper by Knol and Wanderweele (Recommendations for presenting analyses of effect modification and interaction, Int J Epidemiol. 2012). This paper may be regarded as an extension of the extended STROBE guidelines. On line #254 – 258 we also state that the test for a difference in slope using continuous exposure variables and stratified groups was to provide a complimentary test of interaction. In the revised manuscript, we decided to avoid the word “effect modification”. In tables S4 and S5, we simply state “Interaction term” and specific below that we test for a difference in slope.

   Quoting:
    #223 – 233. The first objective was to assess the separate associations of mental health problems and of alcohol intake with all-cause and CVD mortality. To address this, we used Cox Proportional Hazard Models to estimate hazard ratios (HRs), 95% CIs and p-values according to the mental health index and according to alcohol intake (separate analysis). To address the second objective of a potential interaction, we present HRs for their association within strata of each other (stratified analysis) and for their joint association by using a common reference category (joint analysis). This set-up provides the information required to interpret the interaction from different perspectives, the influence from covariates, and to perform recalculations [26, 27].
3. **Use of nonstandard language: throughout, the papers uses somewhat nonstandard language (e.g., referring to "risks" and "risk differences" to discuss smoothed hazard plots). I recommend staying away from these terms in this context.**

   We have gone through the manuscript and supplemental data to remove nonstandard language. Especially the use of the word risk difference was incorrect. We also detected and error in Figure 1 were we wrote Log hazard rate and not log hazard ratio. In the result section, we now mostly refer to the HRs and not risk. Smoothed plots are now referred to as “plots showing the functional form of the associations”. The word risk is used when stating objectives and when discussing the results.

Minor comments:

1. **Abstract: were expected HRs under interaction based on an assumption of multiplicative or additive interaction.**

   In the revised manuscript, in the abstract we now clearly states that the test for interaction was based on an assumption of a multiplicative interaction.

   Quoting:
   #48-51: “HRs for the combination of a high index score and high intakes (HRs: 2.29 for all-cause and 1.78 for CVD) were 64% (95% CI: 53 – 74%) and 69% (42 – 97%) higher than expected under the assumption of a multiplicative interaction structure”.

   In the revised manuscript, we now also give 95% confidence intervals around the estimate of interaction estimated from standard errors obtained with the delta method. This is mentioned in the method section under “Statistical analyses” #253-254.
2. **Line 82: a figure would be helpful to understand this complex design. As written, it is a bit unclear who the target population is and who the study generalizes to. Are any important groups excluded or overrepresented?**

   In the revised manuscript, we have made changes in the paragraph “Study population, data linkage, ethical approval, and selection” to make the study design and the target and source population more clear.

   Quoting:
   #119-129: “The Age 40 Program is a collection of cardiovascular health surveys that invited all men and women aged 40 – 42 years in 13 out of 19 counties in Norway to participate. The Cohort of Norway (CONOR) is a collection of population-based cardiovascular health surveys from different geographical areas in Norway, both rural and urban, where various age groups were invited [21]. To conduct this study, we identified surveys within the Age 40 Program and CONOR that measured both alcohol intake and mental health problems. The surveys identified were performed between 1994 and 2002, had a response rate ranging from 37.5 – 78%, and constitutes a source population of 307,541 visits where both sexes are rather equally represented and where approximately 73% were in the age range 35 to 50 years when attending (Tab S1).

   Generalizability is now discussed more in detail in the discussion in the paragraph “Methodological considerations” #503-520. This discussion also needs to include the individuals excluded for missing values. We think that these changes provides the manuscript with the information the reviewer was asking for.

   In brief, we think there are good reasons to believe that more frail individuals, people with more extreme intakes of alcohol, and people with more severe mental health problems could be underrepresented in the study population.
3. **Line 113: was the categorization of the index based on the mean score per question?**

   Yes, and thank you. The revised manuscript clearly states that the categorization of the mental health index is based on the mean score per question.
4. **Line 168: Here, or early in the statistical analysis section, I recommend stating clearly which hazard ratios were estimated and why.**

   We have revised the paragraph to better communicate the flow of the statistical analyses and the purpose of the different analyses. Which hazard ratios were estimated and why is now introduced more clearly at top of the paragraph. We hope that this was what the reviewer was suggesting. Please also see our reply to the major comment number 2.
5. **Line 168: What was the timescale for the hazard ratios?**

   In the revised manuscript (#227-228), we inform that the timescale used in Cox models was time-on study (days), estimated from the date the individual attended a health survey and until death, emigration, or censoring. The models in the previous manuscript all included age at entry as a covariate, but the revised manuscript also contains unadjusted results without age at entry as a covariate as requested by the editors.
6. **Line 170: Is there a conceptual model or causal diagram that informs which variables are considered mediators vs confounders? I would have expected biomarkers and current health indicators (heart rate, diabetes, serum cholesterol and serum triglycerides) to be on the causal path (ie play a mediating role) as well.**

   Thank you for this comment. We see that the previous version of the manuscript did not provide enough details regarding this. The details provided were also not entirely clear. In the revised version, we specify that blood pressure and HDL-C were considered as potential mediators of the relationship of alcohol intake with CVD and all-cause mortality. The variables included in the multivariable model were considered more likely to be confounders (and thus included). However, this was specific to alcohol intake as the exposure. In terms of mental health as an exposure, the reviewer mentions several variables that may very well be on the causal path. We state that the results of the age and sex adjusted model should be interpreted more in line of a liberal effect estimate of relationships involving the mental health index, while the multivariable adjusted model is a more conservative approach that could be an underestimate. We think that this is a humble approach with respect to the complex exposure in hand. Please see the paragraph “Statistical analysis” and #233-242. When discussing the results involving mental health, we repeat this point (paragraph “Mental health and mortality” and #444 – 451). We also mention in the last paragraph in the discussion that a study using repeated measurements could provide more answers.
7. **Line 175: The paragraph that starts at this line should clearly state which hazard ratios were estimated to address which scientific hypotheses. As written, it is unclear to me why so many different types of results were presented. For example, why present both the interaction terms with the continuous variables and the joint reference category HRs?**

   We agree with the reviewer. Please see our reply to the major comment number 2 which overlaps with this comment. Below we elaborate some more.

   The reviewer asks why we, in addition to the categorical modeling, provide stratified HRs using a continuous exposure variable and test for a difference in slope. The answer to this question is that we think it could also be informative to the reader in terms of interpreting the interaction. For a reader most interesting in what the interaction implies for the association between mental health and all-cause mortality, the stratified analyses, and perhaps the continuous measure with a difference in slope, could be more informative than the joint association. The joint association for example does not show how the role of adjusting for confounding on the separate association with mental health. However, we do consider the joint HRs and the test for interaction using those risk estimates as the primary analyses, and we have made it clear that the test for a difference in slope is a complement. If the reviewer strongly disagrees with our decisions to include these analyses, we are willing to reconsider.
8. **Line 181: Please define a, b, and c in this equation.**

   In the revised version, we have tried an another way of describing how we test for interaction. We hope that the alternative explanation is clearer. Please see the paragraph “Statistical analysis” in the method section.

   Quoting:
   #246 – 254: “Based on HRs from the joint analysis, we tested for interaction by assessing whether the observed joint HR differed from the expected joint HR using a multiplicative interaction structure. We did this for all possible combinations of the exposure categories. Specifically, we divided the observed joint HR for a given combination (moderate index score + moderate alcohol intake) by the expected joint HR, which would in this case be the product of the HRs from A (moderate index score + low alcohol intake) and B (low index score + moderate alcohol intake). Standard errors and 95% CIs were obtained using the Delta method.”
9. **Line 181: it appears that the authors are evaluating whether the joint HR differs from the expected joint HR under a multiplicative interaction structure. Why was this structure chosen rather than an additive interaction structure? (The authors could have evaluated a departure from additivity using the RERI (see Li R, Chambless L. Test for additive interaction in proportional hazards models. Annals of epidemiology. 2007 Mar 1;17(3):227-36 for details)**

   The most important and appropriate measure of interaction in this study is to evaluate the interaction under the assumption of a multiplicative interaction structure, because it provides the best test of the study hypothesis. A test for interaction on the additive scale would ensure that we do not overemphasize the importance of a between-group difference in HR that in absolute terms is very small. We consent that this is very important when presenting group differences related to a drug or a genetic variant, but perhaps less critical for this study, as we know that alcohol intake and mental health problems are responsible for a considerable disease burden. However, we planned to include also a measure of additive interaction, as this was suggested in a paper that we used when deciding on how to perform and present the interactions (Knol and Wanderweele, Recommendations for presenting analyses of effect modification and interaction. Int J Epidemiol. 2012). Unfortunately, there seems to be some issues related to calculating measures of interaction on an additive scale for preventive factors (see the quoted study below). A preventive factor needs to be correctly recoded into a risk factor prior to analyses for the result to be correct. The example the authors use was an easy one (two x two groups), but in our study, we had many categories and had to deal with non-linear relationships. In the light of that amount of uncertainty, and our main focus on multiplicative interaction, we decided not to include the RERI or another measure of additive interaction.


   Quoting from Knol et al. Eur J epidemiol, 2011, Estimating measures of interaction on an additive scale for preventive exposures.

   *“The reason why using preventive factors gives wrong and inconsistent results in the measures of interaction on an additive scale is because a relative risk is restricted between 0 and 1 for a preventive factor while it can go from 1 to infinity for a risk factor. For example, a relative risk of 0.60 means a relative risk reduction of 40%, whereas the inverse (1/0.60 = 1.67) means a relative increase in risk of 67%. Clearly, this could lead to different results if these numbers are used in calculating the measures of additive interaction (e.g. the denominator in the synergy index S could be negative).*
10. **Line 194: Was any of the missing data described in this paragraph likely to be informative? Seems like those missing alcohol data may have been systematically different from those with alcohol data.**

    We have provided a table in the revised manuscript (S2 Table) were we briefly describe the groups of people with missing values. In the main text, we summarize the table in the result section in the paragraph “Study population”.


    Quoting:
    #282-286“Potentially eligible individuals excluded for missing values were on average older, more likely to be male, and died more often from all-causes and CVD during follow-up than individuals in the source population (Tab S2). As a result, the proportion of people dying among complete cases in the study population was somewhat lower than in the source population.”

    We pick up the thread in the discussion in the paragraph “Methodological considerations” were we discuss whether this might have influenced the generalizability of the study findings. See #511-520.

    Quoting:
    *“The study population also deviated from the target population for two reasons. First, the response rate in the health surveys that constituted the source population ranged from 37.5% to 78%. Self-selection into survey attendance likely resulted in a slightly healthier source population than the target population. People with more severe type of mental health problems or more extreme alcohol intake could be underrepresented. Second, the participants excluded for missing values (mostly data on mental health and alcohol intake) were older and frailer than the average participant was. The associations could be different, and possibly more pronounced, in more frail or extreme subpopulations, or in countries where alcoho*l *intakes are higher, the drinking pattern different, or mental health care less available.”*

    A method to deal with missing values include multiple imputation. We have some experience with this method from a previous study, however, on that occasion we only imputed values for missing covariates only. Not the exposure variable, which is indeed a more sensitive subject. The type of missing data becomes more important. Based on a general insecurity about how to deal with the results of an analysis involving multiple imputation of exposure variables, we have not done this. As mentioned above, we discuss what the selection processes may imply for the external validity of the study. We do not state this in the manuscript, but we think it is more likely that the study is underestimating an interaction effect between mental health and alcohol intake on mortality. We are of course willing to reconsider the use of multiple imputation or other approaches for missing values analysis if the reviewer or editor thinks that it is warranted.
11. **Line 199: what proportion of participants were excluded due to missing data?**

    The proportion of participants excluded due to missing data was 17.5%. This was calculated from the number of individuals in the source population, not the number of visits (as there were people who participated in multiple surveys). In the revised version, we state this proportion in the result section paragraph “Study population” #275-276.
12. **Line 203: Including a traditional "table 1" with participant characteristics would help readers follow the paper.**

    In the previous manuscript, the traditional “Table 1” was included as “Table S2” and S3 (for smoking). We made two tables because one table would be too large, but as they were still quite large, we included them in the supplements. In the revised manuscript, we have selected the most important covariates and present them in Table 1. The additional remaining covariates are described in Table S3.
13. **Line 225: In this section, I recommend interpreting the beta coefficients to guide the reader through the analysis.**

    In the revised manuscript, we provide a digested interpretation of what the analysis showed before reporting the exact beta coefficients. We chose to present the beta coefficients with 95% CIs together at the end, and not directly after each exposure category, as the latter would require the use of brackets around brackets. Please see paragraph “Alcohol intake and the mental health index” in the result section line numbers #321 – 328.
14. **Line 230: is the mean presented here the average time of follow-up among those who died or overall? This should be better labeled-**

    We have rephrased the sentence to make it clear that the follow-up period is for all participants. #331-332.
15. **Line 234: How were expected numbers of deaths computed from Kaplan-Meier curves and log rank tests? This was not described in the methods section and is not a familiar approach.**

    We decided to remove the Kaplan-Meier plots and log-rank test from the revised version (please see our comments to major comment number 1).

    But to answer the question. we are not able to reach the same conclusion as the reviewer in that estimating the expected number of deaths is an unfamiliar part of the log rank test. However, we consent that the way we communicated the results might have been awkward or non-standard, or that there might have been some part that was not correct or misleading.

    Quoting from Bland and Altmann, BMJ, 2004, “The Logrank test”:
    *“The logrank test is used to test the null hypothesis that there is no difference between the populations in the probability of an event (here a death) at any time point. The analysis is based on the times of events (here deaths). For each such time we calculate the observed number of deaths in each group and the number expected if there were in reality no difference between the groups.”*

    Exactly how the expected number of deaths is very technical (at least in the authors view), but in practice it seems that the method uses a weighted estimate of the proportion of deaths in the study population overall. We can estimate the proportion of deaths observed in the study population overall (21,376/243,372 ~ 8.8%). Then use this number to get an estimate of the number of deaths among people with a low mean score per question on the mental health index (148,428 = 0.0088 ~ 13,037). This is very close, but not identical, to the expected number produced by the survdif command in the survival package using R statistical software (n = 13,146), which was how we obtained the estimates.
16. **Line 237: the number of CVD deaths was fewer than what? This sentence should be rephrased.**

    We have made changes that removed this sentence altogether.
17. **Line 241 and throughout: These figures present the smoothed hazard function, which is different from the "risk curve."**

    Thank you for commenting on this. The reviewer correctly points out that they are not risk curves. We have discussed the figures with a biostatistician working with survival analysis. The figures were produced using the termplot command in R. It “*plots regression terms against their predictors*”. The predictors were age, sex and the exposure (with a p-spline). The regression terms from the Cox model is log hazard ratios, which is what we presented on the y-axis (except for a typo we have corrected in figure 1, where we in the previous manuscript wrote *rate* and not *ratio*). We write in the “Statistical analyses”:

    *“In the separate and stratified analysis, we also estimated HRs per unit increase of the exposure variables on a continuous scale (for average alcohol intake among current drinkers only), used interaction terms (test for a difference in slope) as a complementary test for interaction, and present the functional form of these associations graphically to support the interpretation of the associations. The functional form was obtained by including the continuous exposure variables as penalised smoothed splines in Cox models adjusted for age and sex. The regression terms (log hazard ratio) were plotted as a function of the mental health index and alcohol intake. The function is centred (log hazard ratio = 0) at the mean value of the predictors..”*

    How to refer to the plots? The reviewer suggest to refer to them as smoothed hazard functions, but the biostatistician thinks that this could also be imprecise, as the hazard functions are a function of time, not the predictors. The biostatistician suggests that we refer to them as “Smoothed log hazard ratios plots”. In the revised manuscript, we merely refer to them as plots depicting the association or functional form of the associations. We have seen this used once before in a publication (de Boer, Serum 25-Hydroxyvitamin D Concentration and Risk for Major Clinical Disease Events in a Community-Based Population of Older Adults, Ann Intern Med. 2012 May 1; 156(9): 627–634.).

    We could manually transform and report the values on the y-axis as hazard ratios (as suggested by a colleague), but because of the log scale, the intervals between for example HR 1.00 and HR 2.00 is much larger than the interval between HR 2.00 and HR 3.00, and so one, and therefore we decided against it.
18. **Figure 1: Methods for producing these figures should be included in the Methods section. In addition, the rationale for producing these figures is not obvious. What hypothesis is being addressed? What do these figures add?**

    We have included the Methods section a description of how they were produced and why. Please also see our reply to comment #1 and comment #17. The main purpose of including the plots was to give the reader a quick overview of the relationships, before we present the result of the formal assessment in the form of the tabulated HRs. The figures are informative about the shape of the relationships (such as whether they are linear or non-linear), curve separation indicates differences between groups or strata, and the degree of CI overlap indicates uncertainty, and the CIs wideness indicate where the number of data points at a given value on the X axis). We have tried to be clear that we do not formally test hypotheses from the smoothed plots. We repeat that we are willing to reconsider the inclusion of these plots if the reviewer thinks that they are redundant.
19. **Line 245 and throughout: This line and below lines refer to the "risk difference" but it is unclear where this is coming from. It appears authors are referring to the Figures, but these figures depict a hazard function. Terms like "risk" and "risk difference" have specific definitions in epidemiology and should be avoided in other contexts to avoid confusion.**

    We apologize for the use of non-standard language and for not providing a clear link between the text and the results. We have followed the reviewer’s suggestion and use HRs throughout in the result section when referring to tabulated result, avoiding the words “risk” and “risk difference”.
20. **Line 307: what is this measure of interaction and how is it defined? Is this the departure from the expected HR under perfect multiplicativity?**

    The reviewer is correct. We have made it clear in the text that we test for interaction under a multiplicative interaction structure.
21. **Tables: Labels in the first column of tables are unclear**

    We apologize for this error. It occurred when embedding the tables in the manuscript text. The labels were there, but hidden because of a lack of row length. We have fixed this in the revised manuscript.
