# Supplementary material for: Association of coincident self-reported mental health problems and alcohol intake with all-cause and cardiovascular disease mortality: A Norwegian pooled population analysis
Source: PLoS Med. 2020 Feb 3;17(2):e1003030. doi: 10.1371/journal.pmed.1003030 (PMC6996806; doi:10.1371/journal.pmed.1003030)
Supplement: Attachment — Submitted filename: Response to reviewers.docx [file pmed.1003030.s015.docx]

# Rebuttal letter

Dear external reviewers and editors at PLOS Medicine.

On behalf of the authors, I would like to express our gratitude for the considerable time that you have invested in the critical appraisal of our manuscript. We are glad that you found the manuscript improved, but understand that not all questions were answered adequately and that new questions emerged in this second round of review. Thank you for allowing us to address them.

In this revision, we have made suggested changes to the abstract and the result section, clarified certain aspects of the study population selection and statistical analyses, and elaborated on the external validity of the results in the discussion. We have also expanded the conclusion sentence in the discussion by stating the public health implications of the findings with respect to alcohol intake among people with mental health problems, as suggested.

We hope that the revised manuscript and the rebuttal letter, which address each specific point made by editors and reviewers, constitutes a further improvement of the manuscript. If you have any further comments or questions, we appreciate the opportunity to address them and make further revisions if needed.

November 27, 2019.

Corresponding author

Eirik Degerud

**Requests from the editors:

Abstract: Methods and Findings: Lines 44-48: Please revise this sentence to: “For CVD mortality, HRs were 0.93 (0.86, 1.00, p = 0.058), 0.90 (0.76, 1.07, p = 0.225), and 0.95 (0.67, 1.33, p = 0.760) for a low index score combined with light, moderate, and high alcohol intakes, and 1.11 (0.98, 1.25, p = 0.102), 0.97 (0.83, 1.13, p = 0.689), 1.01 (0.71, 1.44, p = 0.956), and 1.78 (1.14, 2.78, p =0.011) for high index score combined with light, moderate, and high alcohol intakes, respectively.” to further clarify which analyses correspond to which values.**

We have revised the sentence as suggested, and added “low” for the HR reflecting the combination of a low alcohol intake with a high score on the mental health index (lines 44 – 49).

**Abstract: Background: Lines 27-30: In the final sentence of this section, please add the term “self-reported” (so that it reads “...according to self-reported mental health problems and alcohol intake…”**

We have revised the sentence accordingly (lines 27 – 30).

**Abstract: Methods and Findings: Lines 48-51: Please revise this sentence to: “HRs for the combination of a high index score and high intakes (HRs: 2.29 for all-cause and 1.78 for CVD) were 64% (95% CI: 53 – 74%) and 69% (42 – 97%) higher than expected for all-cause mortality and CVD mortality, respectively, under the assumption of a multiplicative interaction structure.” to clarify which analyses correspond to which data values.**

The sentence has been revised as suggested (lines 49 – 53).

**Abstract: Methods and Findings: Lines 51-52: Please revise this sentence to: “A limitation of our study is that the findings were based on average reported intakes of alcohol without accounting for the drinking pattern.” or similar to clarify the main limitation of the study for the reader.**

We have revised the sentence as suggested by the editors (lines 53 – 55)
 **Results: Lines 326-328: Please specify which beta-coefficient values correspond to which analyses. Also, please provide a reference to the table where the data are presented showing the relationship between mean mental health index score and alcohol intakes (e.g. Table 1).**

In the revised version of the manuscript, we clearly specify which group each beta-coefficient correspond to (lines 336 – 340). We consent that this was unclear in the previous version. A typo was also corrected. The negative (minus) symbol for the 95% CIs for the group “current abstainers” was missing (lines 337 – 338). Based on reviewers 3 suggestion, we simply refer to “regression coefficients”, and not “unstandardized beta coefficients”, in the revised version (lines 277, 336, and 440).

We have also included in the revised version a reference to Table 1 that directs the reader towards the numerical values underlying the lines in the figure (S2 Fig). This is a reference to the crude values. The results of the age and sex adjusted analysis is only given in the manuscript text.

Lines 331 – 332: Quoting: “*The crude relationship between alcohol intake and the mental health index appeared non-linear (S2 Fig and Table 1).”*

**Results: Lines 355-357: Please specify which values correspond to which analyses for abstainers, and light, moderate and heavy drinkers.**

In the revised version, we specify which values correspond to the different alcohol exposure groups and also state the reference category (lines 370 – 373).

**Discussion: Conclusion: Please expand on your conclusion sentence. (For example, you could also speak to the result of your objective to look at the association between alcohol consumption and CVD/all cause mortality broken down by mental health index, and briefly touch on any important clinical implication.)**

In the revised version of the manuscript, we have made some additions to the conclusion sentence (lines 569 – 578):

Quoting:

*“In the general population, the mortality rates associated with more mental health problems and a high alcohol intake were increased when they occurred together. Current low-risk drinking guidelines targeted at the general population state that mental health problems is a reason to consider avoiding or limiting alcohol. The study provides additional observational evidence to support this advice. This potentially involves wide population groups extending beyond diagnosed patients with disorders related to alcohol use or mental health. Clinicians may consider advising patients about the additional harm implicated by this combination even if alcohol intakes and mental health problems jointly or alone do not reach clinical levels of severity.”*
 **S2 Table: Please define the abbreviation “CVD” in the table footnote.**

We have defined “CVD” as “cardiovascular disease” in the table footnote.

**S3 Table: Please define the abbreviations “CVD” and “IHD” in the table footnote.**

We have defined “CVD” as “cardiovascular disease”, “IHD” as “ischemic heart disease”, and “HDL-C” as “high density lipoprotein cholesterol” in the table footnote.

**Figures 1 and 2: Please explain why twice the SD used rather than 95% CIs.**

In the revised version, we write in the legend of Figure 1 (lines 358 – 363) and Figure 2 (lines 428 – 433) the following:

*Quoting:*

*“The solid line depicts the spline and the dashed curves depict twice the standard error (a close approximation of a 95% confidence interval, as 95% of the values fall within 1.96 * standard error of the mean if the values are normally distributed).* *The termplot function in R statistical software was used for visualisation and the function plots twice the standard errors.”*

Comment: For researchers using R statistical software to analyse their data (which we did), there are several functions (from different packages) available for visualising the function form of the relationship between a given exposure (when fitted with spline functions) and an outcome from a multivariable Cox Proportional Hazard model. They produce indistinguishable confidence bands, but the curves are centred differently depending on which package you chose and there are also differences in other options. We have used the function called “*termplot*”, which is included in the base installation of R in the *Stats* package, which is maintained by the R Core Team. Accordingly, it’s an established procedure and has for example been used for the same purpose previously (*de Boer et al, “Serum 25-Hydroxyvitamin D Concentration and Risk for Major Clinical Disease Events in a Community-Based Population of Older Adults”, Ann Intern Med. 2012 May 1; 156(9): 627–634*.). Twice the standard error is a close approximation to a 95% confidence interval, because if the value is normally distributed, then 95% of them fall within 1.96 * standard error of the mean. It's that 1.96 that 2 is a close approximation of. We hope that the clarification in the figure legend provides sufficient information.

**Supporting Information: S1 protocol: Please provide a descriptive title for this item.**

In the revised version, we have included the following descriptive title for the S1 Protocol (lines 733 – 736):

*“S1 Protocol. This study is part of a larger research project with a protocol containing information about the planned project, including background, research questions, most of the data sources, main variables, and pre-planned data analysis and study samples.”*

**Comments from the reviewers:

Reviewer #2: The authors have provided a detailed and clear account of the revisions made to this manuscript in their rebuttal. I have focussed on the general comments I made on the initial submission and the responses to my five specific points. Overall, I will leave the other reviewers to address the revisions made in response to their points but I have checked specific things they raised that were related to my own initial concerns, particularly Reviewers 1's point about psychotropic medication and Reviewer 3's first two points. I am very happy with the responses made by the authors to my original review and find the manuscript to be considerably improved. For the second of my specific points, I agree that little purpose would be served by including the sensitivity analysis with a limited age range in the final manuscript. The proportion of the sample aged under 40 at initial attendance is too small to make a notable difference.
There are some minor issues with the language of the manuscript. Some examples I remember were:
line 283 - "and died more often from all-causes and CVD" would read better as "and more likely to have died from all-causes and CVD";
line 449 - "However, the study design prevent further investigation" should be "However, the study design prevents further investigation";
line 537 - "Such as study" should be "Such a study".**

We are very glad that the reviewer found the manuscript improved with respect to own comments and the related comments made by other reviewers. We thank the reviewer for also pointing out the errors in the language and have corrected them and others identified throughout the manuscript.

**Reviewer #3:**

**Overall, this revision was very responsive to my previous comments. A**

**few issues remain and are outlined below.**

**Major Comments**

1. **Thank you for providing detailed information about the collection of surveys that gave rise to the study data. However, I still do not understand how the one visit per individual was chosen (as mentioned on line 130) if individuals had more than one “visit” in any of the data sources? This is a concern because differences in the timing of selected visits by exposure group could confound the results (which is one reason to control for age, as the authors point out). This should be clarified.**

   We apologize for not providing a clear description of this in the previous version. It could be that the lack of clarity might have given the impression that most of the individuals in the study population attended several surveys and were thus represented in the source population by several visits. This is not the case. The vast majority only attended a single survey, and when we write that we “selected one visit per individual”, this was the only visit/observation/ survey attendance available for the vast majority.

   The reason why we have to “select one visit per individual” is because a small proportion did attended several of the source surveys and were represented by more than one visit in the source population. The reason why people attending several of the source surveys was coincidental for the small overlap between surveys in CONOR and the Age 40 Program, and we argue that placing an arbitrary priority on surveys in CONOR should not be a cause for concern. The overlap was not coincidental for individuals who attended the fourth and fifth part of the Tromsø study (within CONOR). In the Tromsø study, people who attended one survey was re-invited to attend the next survey alongside new participants who have never attended before. We chose to include the first visit for individuals who attended both Tromsø 4 and 5, which included approximately 7000 individuals. There could be arguments for including the second over the first, and vice versa, but we did not find any convincing evidence for either and therefore chose the first.

   In the revised version of the manuscript, we have provided the details above in a more condensed form. Please see lines 132 – 143. We also hope that this provides the reader with clear information about how multiple visits where handled in the selection process, and that this was not the case for the vast majority of the individuals in the source population. We also hope that this is reassuring with respect to confounding due to the selection of multiple visits.

   We agree that if we had placed priority differently, there is a theoretical possibility that the non-selected visits had very different measurements of alcohol intake and mental health, and possibly the associations with all-cause and CVD mortality could differ. However, without convincing arguments that the current selection process is something that is wrong or sub-optimal, and in light of the size of this subset, we are reluctant to make any changes to the selection at this stage.
2. **How were covariates included in the regression models? For example, was age modeled as a linear variable, using categories, or another type of flexible functional form? How were functional forms chosen?**

   In the revised version of the manuscript, in the method section and the paragraph “Statistical analysis”, we now include information that describes in what format the covariates were included in the regression models (lines 246 – 247). Age, body mass index, serum cholesterol, and serum triglycerides were modelled as linear variables, while the other covariates were modelled as categorical variables.

   We consent that we did not consider other flexible forms when choosing how to include the covariates in the regression models. This should have been done for age, which is an important covariate, and we thank the reviewer for this comment. We have performed a range of sensitivity analyses during this revision process were we compare the result of fitting age as a linear variable, a second order polynomial, or using penalised splines. The sensitivity analyses show that the regression coefficients for other covariates and the exposures do not materially differ depending on how age is modelled with respect to these functional forms. The analyses also suggest that adding age as a linear predictor is appropriate. We have not included any mentioning of the sensitivity analyses in the revised version, as this is how we interpret the reviewer’s comment. However, we will of course reconsider if the reviewer thinks that information should be added.

**Minor points**

1. **Line 239: the paper notes that HDL-C and blood pressure were considered mediators of the alcohol-mortality relationship. The following sentence seems to indicate that these variables were considered confounders of the mental health-mortality associations, and therefore included in the models for mental health. How were these variables handled in the models for the joint effects?**

   We apologize for not communicating clearly. In the revised manuscript, we state clearly that the unadjusted model, the model adjusted for age and sex, and the same multivariable model was applied to assess the separate, joint, and stratified associations involving alcohol intake and mental health (lines 242 – 246). We also corrected an error by adding BMI to the list of potential confounders in the manuscript text BMI was listed in the Tables, but had fallen out from the manuscript text. Furthermore, we state clearly that the added covariates were considered confounders of the associations involving alcohol intake and associations involving mental health (lines 248 – 249). We did not specify this in the previous version and we thank the reviewer for pointing this out. With regards to mental health problems and the role of confounding/mediation, please see our reply to the next question (minor comment #2).

   Regarding HDL-C and blood pressure:
   For associations involving alcohol intake, we considered these variables to be potential mediators, as the reviewer points out. This is because evidence from observational studies, short-term RCTs, and Mendelian randomization studies suggest that they are directly influenced by a change in alcohol intake. Theory therefore suggest that they should be excluded from the multivariable model when applied to the separate analysis. We also excluded them from the joint assessment, although we consent that we were lacking information on how to handle a potential mediator of one exposure and potential confounder of another exposure in a situation where you want to estimate the joint HRs. However, we controlled that including systolic blood pressure did not materially alter the joint HRs.

   As we had to exclude blood pressure and HDL-C from the separate models involving alcohol and also the joint assessment model, were including it was trivial in terms of the associations of interest in this study, we consent that we were motivated by the possibility to fit the same multivariable model in all the analyses. We therefore checked whether including or excluding systolic blood pressure influenced the risk estimates in the separate models involving mental health, and found that it didn’t materially influence the HRs. HDL-C was missing for an additional 16,529 individuals, and thus it was not possible to do the same type of checks for HDL-C, and also the missing values would reduce statistical power. Moreover, there is also little evidence that suggests that HDL-C is causally linked to CVD risk according to the results of Mendelian randomization studies and clinical trials of HDL-lowering drugs. Because of this, we found ourselves in a situation where we had several arguments for applying the same multivariable model for the separate, joint, and stratified assessment of both exposures.

   In the revised manuscript, we have made revision to improve on our reporting. We now provide a brief, but hopefully clear description of how HDL-C and blood pressure was considered and handled in the analyses (lines 249 – 252).
2. **Line 240: the paper states that the multivariable model treats ”these variables” as confounders while the model adjusted for age and sex treats them as mediators. This section is unclear. Do the authors consider these variables to be mediators or confounders? This section should be rewritten to reflect that the scientific question and knowledge of the causal structure determines the models used, and not vice versa.**

   Please refer also to our reply to the previous comment (minor comment #1).

   We agree with the reviewer that the scientific question and knowledge about the causal structure determines the models used. However, knowledge about the causal structure is sometimes not crystal clear and often complex. This is very much the case for mental health, as it harbours the capacity to potentially have direct effects on health (by inducing physiological stress) as well as indirect effects via behavioural changes. The indirect effect could involve many mediators and be, in sum or in another manner, large in magnitude. Treatment of depression may for example lead to obesity not only because of a lack of motivation or incentive to exercise and purchase and cook food healthy food, but also via the side-effects of drugs leading to increased appetite, which could contribute to an increased risk of CVD and mortality. However, obesity could result from non-mental health reasons, and further increase the risk of CVD as well as mental health problems in the context of social stigmatization. This is a source of confounding. Sorting variables into mediators and confounders is therefore difficult in terms of estimating associations with mental health problems, as they could act as both. We tried to communicate this uncertainty in the previous version in the method section.

   In the revised version, we refrain from going into this debate at this stage. We state that we consider the variables to act as confounders on the relationship involving mental health and therefore include them in the multivariable model (lines 248 – 249). This may be regarded as a more conservative approach. The uncertainty regarding whether it is the age and sex-adjusted association, or the multivariable adjusted association, which best reflect the “true” association of mental health with the study outcomes, is something we now only communicate in the discussion:

   *Lines 465 - 470: “A considerable part of the age and sex adjusted association was accounted for by multivariable adjustment, which is in line with the interpretation that mental health may impact mortality via changes in health behaviour.* *However, the study design prevents further investigation into the temporal sequence of such events and it is not possible to establish exactly how much of the association explained that is a result of mediation or by confounding. ”*
3. **Throughout, the paper refers to “beta” coefficients. While this is common language in biostatistics, it may be preferable to refer to regression coefficients to prevent confusion.**

   In the revised manuscript, we have replaced the word “beta” with “regression” at three locations (lines 277, 336, and 440).
4. **In the methods or results, the paper should indicate the maximum follow-up time. In other words, over what time period are the reported hazard ratios applicable? As noted in Hernn MA. The hazards of hazard ratios. Epidemiology (Cambridge, Mass.). 2010 Jan;21(1):13, the hazard ratio is sensitive to the time period chosen.**

   We have included the maximum follow-up time (20 years) in the result section when we give the average follow-up time, which was ~17 years (lines 343 – 344). The follow-up time in years is presented on the x-axis and the frequency on the y-axis on the histogram below. We thank you the interesting paper by Hernan. Please also see our reply to another comment made by the reviewer (#minor comment #6), which is related to external validity.

5. **Line 467: “higher” than what?**

   In the previous version, we wrote “*HRs for all-cause mortality were 9 – 64% higher when mental health problems and alcohol intake occurred together”.*

   In the revised version, we write (Lines 486 – 487): *“HRs for all-cause mortality for the joint association of mental health problems and alcohol intake were 9 – 64% higher than expected under a multiplicative structure”.*
6. **In the “methodological considerations” section of the Discussion, the authors may also want to discuss the fact that, to be included in the study, participants had to survive until mid-life, when the surveys were implemented. Accordingly, results may not hold for the effects of drinking and mental health at earlier ages.**

   We thank the reviewer for this important comment. In the previous version, we did somewhat touch upon the subject.

   Quoting: *“Because of the sampling profile of the health surveys, and the Age 40 Program in particular, the vast majority of the participants in the study population attended a health survey in midlife (73% were between 35 and 50 years of age). A study performed exclusively in younger or older populations, where the nature of non-CVD deaths could differ, may return somewhat different results.”…*

   In the revised version, we elaborate some more to also include the point of survivor bias with a reference to excellent paper by Hernan.

Lines 531 – 535 : *…“* *A related issue is the possibility that individuals who were the most vulnerable or susceptible to the negative effects of alcohol or mental health problems could have been more likely to have died prior to the health surveys, which could result in underestimation of the risk associated with alcohol intake and mental health problems [38]..”*
