# Supplementary material for: Association of coincident self-reported mental health problems and alcohol intake with all-cause and cardiovascular disease mortality: A Norwegian pooled population analysis
Source: PLoS Med. 2020 Feb 3;17(2):e1003030. doi: 10.1371/journal.pmed.1003030 (PMC6996806; doi:10.1371/journal.pmed.1003030)
Supplement: Attachment — Submitted filename: Response to reviewers.docx [file pmed.1003030.s016.docx]

**Production issues:

Figures

-       Please note that Fig 2 is currently split into multiple parts in separate files. Please either combine the parts into one figure with lettered panels or renumber your figures as whole parts (i.e. Fig 1A becomes Fig 1, Fig 1B becomes Fig 2, etc.), ensuring that all relevant legends and in-text citations have been updated accordingly.**

In the revised manuscript, we have combined the parts into one figure with lettered panels. We have included A over B, but could, on request, place them side by side.

**Metadata

-       Please ensure that the CRediT author contributions listed under each co-author are completed in full. To qualify for authorship, each author must meet at least one of the seven core contributions (conceptualization; data curation; formal analysis; investigation; methodology; software; validation), as well as at least one of the writing contributions (original draft preparation; review and editing). Authors may also be noted against any of the remaining contributions, however these alone will not qualify a contributor for authorship.

Author Luisa Zuccolo is noted as contributing to a writing role only, which, as mentioned above, does not qualify them for authorship. Please ensure that the full contributions of each author are acknowledged in the "Add/Edit/Remove Authors" section of our submission form. If they do not meet criteria for authorship please let us know as soon as possible by emailing** [**plosmedicine@plos.org**](mailto:plosmedicine@plos.org)**.
-       In accordance with PLOS' data policy, please ensure that your Data Availability Statement in the submission form clearly identifies how readers can access your data. Note that it will be typeset as it is written so please ensure it is in complete sentences and appears as you would like it to in the published version.

For details on our data policy, please see** [**http://journals.plos.org/plosmedicine/s/data-availability**](http://journals.plos.org/plosmedicine/s/data-availability)

Thank you for making us aware about this error. We have corrected the Author Contribution roles for Luisa Zuccolo to include both “Writing and editing” as well as “Conceptualisation”.

**Article body

-       Please remove the funding information from your manuscript file. The published version is pulled automatically from the “Financial Disclosure” field in the submission form and must not be duplicated. Please therefore take this opportunity to check that the information in this field is correct and completed in full.**

We have removed the funding information from the manuscript file and checked the information in the submission form.

**-       Please remove the Competing Interests section from your manuscript file. The published version is pulled automatically from the "Competing Interests" field in the submission form and must not be duplicated. Please therefore take this opportunity to check that the information in this field is correct and completed in full.**

We have removed the Competing Interest section from the manuscript file and checked the information in the submission form.

**-       Please remove your Author Contributions section from your manuscript file as this information is automatically pulled from the authors section of our submission form and must not be duplicated. Please therefore take this opportunity to check that the information in this field is correct and completed in full.**

We have removed the Author Contributions section from the manuscript file and checked the information in the submission form.

**-       Please ensure that your Ethics statement is available in the Methods section of your manuscript in its entirety and not at the end of the manuscript.**

In the revised manuscript, we made a small revision to the Ethics statement in the Method section. We also deleted the statement at the end of the manuscript, which was a duplicate.

**-       Please remove the data availability information from your manuscript file as this is automatically pulled from the "Data Availability" field in the submission form and must not be duplicated. Please therefore take this opportunity to check that the information in this field is correct and completed in full.**

We have removed the data availability information from the manuscript file and checked the information in the submission form. We also made revision to this statement, as suggested by the Editors.

**Tables

-       Please ensure that all main tables in their entirety (including titles and footnotes), are placed after the paragraph containing their first in-text citation.**

We think this comment was referring to Table 1. In the previous manuscript, there was a paragraph between the paragraph containing the first in-text citation of Table 1 and Table 1. In the revised manuscript, we have merged these two paragraphs. It should be ok, now.
 **References

-       Please put all reference citations in square brackets separated by either commas or dashes with no spaces, e.g. [1,2,3] or [1-3].
-       Please ensure your references are in the style of PLOS Medicine (ICMJE/Vancouver style). The reference style guide can be found here:** [**http://journals.plos.org/plosmedicine/s/submission-guidelines#loc-references**](http://journals.plos.org/plosmedicine/s/submission-guidelines#_blank)**. You can also find a downloadable EndNote file in these guidelines to assist you. Please do a manual check as well, as the EndNote file does not catch everything. Please note that the references will be typeset as they appear so please ensure the format is consistent.**

Based on the above comments, we made changes to the output style in Endnote for PLOS that we had used in the previous version of the manuscript. We removed the “white space” in the [1-3], but found no “white space” in the [1,2,3]. We have checked and this seems ok now. We also removed the automatic output for PUBMED central, which was included in the previous version. We hope that the references are ok now.

**Supporting Information

-       Please ensure that all Supporting Information files use the following format verbatim throughout the manuscript (including legends and all in-text citations): S1 Fig, S1 Table, S1 Text, S1 Data, etc. Each file type should start at 1 and increase numerically. Please note, however, that the file names themselves must use an underscore rather than a space, i.e. S1_Fig.tif, S1_Table.xlsx, S1_Text.doc, S1_Data.xlsx, S1_PRISMA_Checklist.docx, etc.**

We made three corrections (S1 Fig, S3 Text, and S1 Table) in the manuscript text in the Method section.

**Requests from Editors:

1. Data Availability Statement:  Thank you for providing your data availability statement and the contact information for access to your study’s data.  Please remove the following text from the statement, as this detail is not needed: “The reason why the data are not made publicly available in a public repository or as a supporting information is because of local legal restrictions as well as ethical restrictions related to privacy. The participants have not consented to their data becoming publicly available and they might lose control of their data, such as their right to have their data deleted.”**

We have removed the redundant text, as suggested, in the Data Availability Statement in the submission form.
 **2. Title: Please revise your title according to PLOS Medicine's style. Your title must be nondeclarative and not a question. Please place the study design in the subtitle, after a colon rather than a hyphen.  When you revise your title, please update the text of the manuscript as well as the manuscript submission form with your revised title.  We suggest, “Association of coincident self-reported mental health problems and alcohol intake with all-cause and cardiovascular disease mortality: A Norwegian pooled population analysis” or similar.**

We like the suggested title and have revised the title accordingly in the manuscript text and the submission form.
 **3. Abstract: Methods and Findings: Line 36: Please indicate here that alcohol intake was “self-reported”.**

We now indicate that alcohol intake was self-reported (Line 39).
 **4. Abstract: Methods and Findings: Please provide some summary demographic information for the study participants, e.g. prevalence information, mortality, etc.**

In the revised manuscript, we have included the following demographic information for the study participants.

Quoting (line number 33 – 36): “*The mean (standard deviation) age at the time of participation in the survey was 43.9 (10.6) years and 47.8% were men. During a mean follow-up period of 16.7 (3.2) years, 6587 participants died from CVD, and 21,376 died from all-causes.”*
 **5. Abstract: Methods and Findings: Line 42: Please revise to “...light, moderate, and high intakes, respectively.”**

We have made the suggested correction (Line 45).
 **6. Abstract: Conclusions: Please revise this sentence to clarify “they” in “...were increased when they occurred together.”**

In the conclusion in the abstract and in the main text, we now write “...when the risk factors occurred together” and not “they” (Line number 59 – 60).
 **7. Author summary: Please use bullets rather than dashes for separate points.**

We now use bullets, not dashes.
 **8. Author Summary: Why was this study done?:  Please revise the second point to clarify, we suggest: “Many people both drink alcohol and experience mental health problems, but we do not have much data showing whether the combination of drinking alcohol and mental health problems is associated with additional negative health consequences.”**

We have revised the sentence as suggested by the Editors.

**9. Author Summary: What did the researchers do and find?: Please clarify the second point, we suggest: “The risk of all-cause and cardiovascular disease mortality was higher among people defined with more mental health problems and a high alcohol intake (“>24 g/day”) than would be expected for the linear combination of high alcohol intake and high mental health index.”**

We have revised the sentence as suggested by the Editors.

**10. Author Summary: What do these findings mean?: Please clarify the bullet points and remove causal language, similar to this, according to your intended meaning:
-  The findings suggest that co-occurring alcohol intake and mental health problems are associated with increased negative health effects including all-cause and cardiovascular disease-related mortality.
-  Our findings may help to inform clinical recommendations regarding potential risks of alcohol use by individuals with mental health problems.
- The findings warrant future studies with longitudinal data that can shed more light on the mechanisms underlying the interaction between alcohol intake, mental health, and mortality.**

We have clarified the bullet points as suggested by the Editors. We made a small addition to the second point (line number 84 – 86).

“Our findings may help to inform clinical recommendations *and low-risk drinking guidelines* regarding potential risks of alcohol use by individuals with mental health problems.”

**11. Introduction: Line 104: Please change “relation” to “relationship”.**

We changed the word “relation” to the word “relationship” on two occasions in the last paragraph in the introduction (line numbers 112 and 118), as well as once in the “Abstract - Background” (Line 25)**.


12. Introduction: Lines 106-108: The meaning of the word “trait” is not clear, please clarify these sentences.**

We have revised the sentences were we used the word “trait” in the introduction and discussion. We no longer use the word, and hope that the content in the sentences are now communicated clearly (line number 112 – 116).

**13. Introduction: Lines 104-111: Please replace “relation” with “relationship”.**

We changed the word “relation” to the word “relationship” on two occasions in the last paragraph in the introduction (line numbers 112 and 118), as well as once in the “Abstract - Background” (Line 25)**.


14. Introduction: Line 118: Please change “interaction” to “an interaction between mental health problems and alcohol intake.” or similar, according to your meaning.**

We revised the sentence as suggested by the Editors here and in comment number 15 (lines numbers 124 – 127).

**15. Introduction: Final sentence: Please revise to reduce causal language: “The second objective was to investigate whether risks of all-cause or CVD-related mortality are increased in persons who report mental health problems and alcohol intake in combination, suggesting an interaction between mental health problems and alcohol intake.” or similar**

We revised the sentence as suggested by the Editors here and in comment number 14 (Line numbers 124 – 127).

**16. Methods: Line 131: Please clarify if by “when attending” you mean “at the time of participation in the survey”**

We have clarified the sentence as suggested (line numbers 136 – 140).
 **17. Results: Lines 282-289: Please clarify that the 51,754 participants excluded is the total excluded, and you then discuss the breakdown of that number by type of missing data.**

We have made revisions that clarifies that 51,754 represents the total number of participants excluded and that the additional information is a breakdown of the type of missing data (line numbers 294 – 299).
 **18. Results: Lines 342-343: Please give the minimum follow up time, as well as the maximum.**

We have included the minimum follow-up time together with the maximum. The minimum follow-up time was 0 days (line numbers 351 – 352).

0 days may seem a bit odd, but one has to keep in mind that this is a large observational study were people attended surveys at different ages. There is no apparent reason to exclude people who died in a short period after baseline, such as in a follow-up study of patients undergoing some invasive surgery etc.

For transparency, this is the number of days of follow-up for the participants with the shortest follow-up time (limited to 100 people).

```
> head(sort(d1$dager_out), n=100)
  [1]  0  0  1  3  4  6  7  8 12 13 13 14 14 15 17 18 20 22 23 24 25 27
 [23] 28 29 32 32 33 34 34 35 35 35 36 36 38 39 42 43 43 44 44 45 45 45
 [45] 46 46 47 48 48 48 49 49 50 52 53 53 54 55 55 56 56 56 56 57 60 60
 [67] 60 61 64 66 66 69 69 70 70 71 72 72 72 74 74 75 75 75 75 75 75 76
 [89] 78 81 81 81 81 85 85 86 86 86 87 88
```

This is the number of days of follow-up for the participants who died and who had the shortest follow-up time (limited to 100 people)

```
> head(sort(d1$dager_out[d1$DOD==1]), n=100)
  [1]   0   1   6  12  13  13  14  14  17  18  20  22  23  27  28  29
 [17]  32  32  33  34  34  35  36  36  39  42  43  43  44  45  45  46
 [33]  46  48  48  48  49  49  52  53  53  54  55  55  56  56  56  57
 [49]  60  61  64  66  69  70  72  72  72  74  74  75  75  75  75  75
 [65]  78  81  81  81  81  85  85  86  86  86  87  88  88  88  89  90
 [81]  92  92  93  94  94  95  97  97  98  98  99  99 100 100 101 101
 [97] 102 102 103 106
```

**19. Results: Lines 366-372: Please provide p values for analyses of alcohol intake and the risk of all-cause and CVD mortality.**

In the revised version of the manuscript, we have included p-values for the analyses of alcohol intake and the risk of both all-cause and CVD mortality (line numbers 376 – 383). **20. Results: Lines 380-386: Please clarify what is meant when you say “higher” or “higher than expected” by providing the reader with information on what would be “expected” and also describing the HRs you observed, and demonstrating how they differ (with 95% CIs and p values).**

In the revised version, we have tried our best to guide the reader through the interaction analyses involving the joint analyses (line numbers 387 – 411). We mention which combined groups, as well as give HRs (with 95% CIs and p-values), that were used to calculate the “expected” HRs under the assumption of no interaction. We then provide the observed joint HRs and HRs for the interaction, both with 95% CIs and p values. For clarity, we underscore that the “expected joint HR” by itself is not applicable with a CI or p value.

We provide all these details when we refer to the results involving all-cause mortality and the combined categories of different alcohol intake levels with a high score on the mental health index. When we later in the paragraph refer to the results involving all-cause mortality and the combination of alcohol intake with a medium score on the mental health index, as well as results involving CVD mortality, we provide the joint observed HR and the HR for the interaction (with 95% CI and p-values). We also provide the expected joint HR and give the HRs used to calculate it, but do not go to the length of stating which combined categories were used, as this becomes very lengthy. By now, we assume that the reader has obtained an understanding of which groups are used to calculate the expected joint HRs for a given combined category, and when aided by the HRs given, should easily find the groups in Table S4. If the editors disagree, we are willing to reconsider this.

 **21. Results: Lines 406-410: Please provide the 95% CIs and p values for the relationships described here.  In the sentence at line 406 please clarify that this describes the relationship for all-cause mortality.**

In the revised version, we have added the HRs with 95% CIs and p-values for the relevant categories and for the test for slope difference (line numbers 431 – 438).

Quoting:

“In stratified analyses, a one-unit increase in mean score per question on the mental health index was consistently associated with a higher HR for all-cause mortality at all alcohol intake levels (S4 Table). The point estimates and test for slope difference indicated a more pronounced change in HRs for all-cause mortality among people reporting moderate and high alcohol intakes versus low intake. HRs for all-cause mortality per unit change in the mental health index in the low, moderate and high strata were 1.15 (95% CI: 1.10, 1.21, p < 0.001), 1.32 (95% CI: 1.17, 1.48, p < 0.001), and 1.32 (95% CI: 1.17, 1.48, p < 0.001), respectively. The tests for a slope difference were 1.22 (95% CI: 1.08, 1.38, p = 0.002) and 1.38 (95% CI: 1.16, 1.63, p < 0.001).”

**22. Results: Lines 418-420: Please clarify what is meant when you say “test for a difference in slope was positive”.**

In the revised version of the manuscript, we have changed the sentence to the following to clarify the meaning and also added the estimates for the interaction..

Quoting (line numbers 448 – 452):

“The result of the test for slope difference was 1.07 (95% CI: 1.04, 1.10, p < 0.001) for all-cause and 1.10 (95% CI: 1.04, 1.16, p = 0.001) for CVD mortality, indicating a more pronounced change in HRs per unit change in alcohol intake among people a high vs low score on the mental health index.” **23. Discussion: Line 540: Please clarify the term “frailer”.**

We have made revisions that hopefully clarifies the use of the term “frail”. If there are still issues with the term, we will find an alternative.

Quoting (line numbers 572 – 577):

“Second, the participants excluded for missing values (mostly data on mental health and alcohol intake) were older and were more likely to have died during follow-up than the average participant was, suggesting that they might have been more frail. The associations could be different, and possibly more pronounced, in more frail or extreme subpopulations, or in countries where alcohol intakes are higher, the drinking pattern different, or mental health care less available.”

**24. Discussion: Line 546-548: Please revise this sentence to avoid causal language, we suggest: “This study suggests that in a large sample of the general population that mortality risk is increased in people who report more mental health problems and a higher alcohol intake, raising the possibility of interaction between risks associated with mental health problems and higher alcohol intake.” or similar.**

We thank the Editors for this suggestion. We have revised the sentence to the following:

Quoting (line numbers 580 – 583):

“This study was performed in a large sample of the general population. The findings suggest that mortality risk is increased in people who both report more mental health problems and a higher alcohol intake, raising the possibility of interaction between risks associated with mental health problems and higher alcohol intakes.”

As repeated below, we hope that the revised manuscript is purged from causal language.

**25. Discussion: Lines 548 and Line 572 (and throughout): Please revise to avoid the use of causal language (“evidence”) as your study is observational and causality cannot be suggested.** 

We have gone through the manuscript and made several minor changes throughout, resulting in a revised manuscript that hopefully is free from using causal language, such as the word “evidence”. We hope that there are no more remaining instances where causality is suggested, as it was never our intent to suggest in those lines. We are well aware of the limitations of this observational study.
 **26. Discussion: Conclusions: First sentence: Please add “Our study found…” or similar to the sentence.**

We have revised the sentence to start with “Our study found...” (line 604).
 **27. Discussion: Conclusions: Line 572-574: This sentence is not clear, please revise: “This potentially involves wide population groups extending beyond diagnosed patients with disorders related to alcohol use or mental health.”**

In the revised manuscript, we have deleted the sentence completely. We consider the content in this sentence to be conserved in what was the following sentence in the conclusion.

Quoting (line numbers 608 – 611):
“Clinicians may consider advising patients about the additional harm implicated by this combination even if alcohol intakes and mental health problems jointly or alone do not reach clinical levels of severity.” **28. Figure 2 legend: Please clarify what is meant by “functional forms” in the title.**

During the peer-review process, based on comments from reviewer 3, we discussed what to call these figures (Fig 2). There were several suggestions. The use of “functional form” was used, because it has been used previously by others. Functional form refers to the spline function used to model and visualise the exposure variable.

The purpose of showing the figures is to give the reader something visual or graphical to help him or her get a quicker overview than what the different tables provide. In the revised version, we now refer in the title of both Figure 1 (line 362) and Figure 2 (line 454) that they are “Visualisations of the associations...” and do not use “functional form” or any other technical word. We think this works better. The manner in which they are produces are explained in detail in the figure legend below the figure title. We have also made changes in the manuscript text at the places where we refer to Figure 1 (line 353) and Figure 2 (line 442). We only write “functional form” once in the paragraph “Statistical analysis”. We hope that the editors approve of these changes.
 **29. Table 1 Legend: Rather than saying a high score is unfavorable, please clarify specifically what a high score indicates (e.g. indicates greater numbers of self reported mental health issues).**

In the revised version, we no longer say that a high score is unfavourable. We do as the Editors suggest and say that it indicates greater numbers of self-reported mental health issues (lines 335 – 336).  **30. Table 2 and Table 3: Please present both the 95% CIs and p values for the interaction effects.**

In the revised version of the manuscript, in Table 2 and Table 2, we have calculated and present p values together with the HRs and 95% CIs. We also provide the p values in the manuscript text, where applicable.
 **31. S2 Figure: Please clarify in the legend the grey shading represents the 95% CIs.**

In the revised manuscript, we clarify that the grey shading represent the 95% CIs in the legend of S2 Figure. We also made small edits to the legend to make it emphasise that the mental health index is the outcome in that analysis (lines 762 – 768).

**32. S1 Checklist: Thank you for including the STROBE Checklist.**

We added the STROBE Checklist with the RECORD extension.
  **33.  Please remove the sections (lines 770 and beyond) on Transparency, Competing Interests, Funding, Ethics Approval, and Data Sharing, as this information is automatically pulled together via the manuscript submission system.**

We have removed the sections from the revised manuscript.
